# Copper-Promoted Hiyama Cross-Coupling of Arylsilanes With Thiuram Reagents: A Facile Synthesis of Aryl Dithiocarbamates

**DOI:** 10.3389/fchem.2022.867806

**Published:** 2022-04-26

**Authors:** Yiying Wang, Hongtao Shen, Jianhua Qiu, Mengqi Chen, Weimin Song, Mingqin Zhao, Longfei Wang, Feng Bai, Hongxia Wang, Zhiyong Wu

**Affiliations:** ^1^ Flavors and Fragrance Engineering and Technology Research Center of Henan Province, College of Tobacco Science, Henan Agricultural University, Zhengzhou, China; ^2^ Technology Center, China Tobacco Henan Industrial Co., Ltd., Zhengzhou, China

**Keywords:** Hiyama cross-coupling, arylsilanes, thiuram reagents, C-S bond formation, aryl dithiocarbamates

## Abstract

We report herein a facile Hiyama cross-coupling reaction of arylsilanes with thiuram reagents (tetraalkylthiuram disulfides or tetraalkylthiuram monosulfide) enabled by copper fluoride. Compared to our previous work, this protocol is an alternative protocol for the generation of S-aryl dithiocarbamates. It features low toxic and readily available substrates, cost-effective promoter, easy performance, and provides good yields.

## Introduction

Transition-metal-catalyzed cross-coupling reactions have been found broad applications for the construction of carbon-carbon and carbon-heteroatom bonds enable the facile preparation of more complex molecules (Miyaura, 2002; Magano and Dunetz, 2011; Negishi, 2011; Suzuki, 2011; Guo and Rueping, 2018; Zhang et al., 2020). In 1972, Kumada and Tamao (Tamao et al., 1972) reported the cross-coupling reaction of Grignard reagents (RMgX) with organic halides (R’X) catalyzed by nickel/phosphine system. From then on, a wide range of organometallic reagents such as lithium (Yamamura et al., 1975; Murahashi et al., 1979), aluminum (Negishi et al., 1978), zinc (Sekiya and Ishikawa, 1976; King et al., 1977; Negishi and Van Horn, 1977; Negishi, 2011), zirconium (Negishi et al., 1977; Okukado et al., 1978) and tin (Milstein and Stille, 1979a; Milstein et al., 1979b) have emerged and exerted a ubiquitous influence on the synthesis community. However, their instability, air and moisture sensitivity and the production of corrosive halogen wastes are disadvantageous from both synthetic and environmental points of view. In addition to these well-established organometallic reagents, the silicon reagent which was developed by Hiyama and co-workers, is an alternative and attractive coupling partner for cross-coupling reactions (the so-called Hiyama cross-coupling) (Nakao and Hiyama, 2011; Sore et al., 2012; Denmark and Ambrosi, 2015; Komiyama et al., 2016). Generally, organosilicon reagents exhibit some remarkable advantages such as non-toxicity, high stability, good tolerance toward various functional groups and natural abundance of silicon. In the overpast several decades, significant advances on transition-metal-catalyzed Hiyama cross-coupling have been achieved (Nareddy et al., 2017; Nareddy et al., 2018; González et al., 2019; Han et al., 2019; Zhang et al., 2019; Idris and Lee, 2020; Lu et al., 2020; Wu et al., 2021), nevertheless, the diverse applications of this methodology are still less explored and worthy of in-depth exploration under the concept of green chemistry.

**SCHEME 1:**
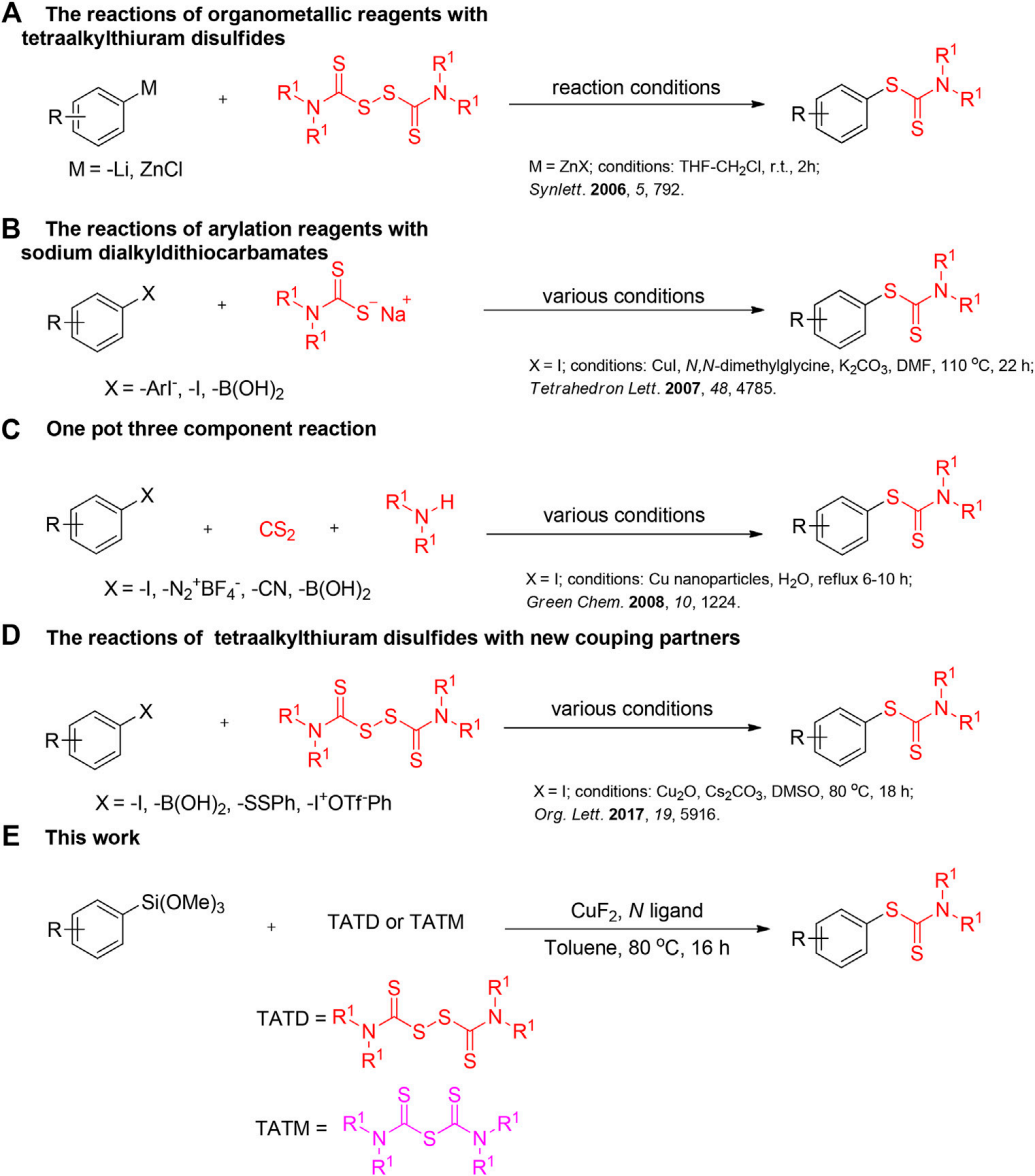
Different methodologies for the synthesis of aryl dithiocarbamates.

Thiuram reagents (tetraalkylthiuram disulfides TMTD, or tetraalkylthiuram monosulfide TMTM) are cheap and stable organosulfur compounds which can be widely used in biologically active compounds, agricultural pesticides and vulcanization accelerators (Enders et al., 2010), and also act as readily available sulfur reagents in organic synthesis. (Zeng et al., 2017a; Zeng et al., 2017b; Wu and Yan, 2019). Among them, organic dithiocarbamates have been extensively investigated for their outstanding biological activities (Hou et al., 2006; Zou et al., 2014; Liénard et al., 2008; Horita et al., 2011) and synthetic value (Boas et al., 2004; Derouet et al., 2009; Wults and Greene, 2007; Zhang et al., 2005). Hence, much attention has been paid to the development of highly efficient and convenient methods for the construction of such scaffolds. Traditionally, the portion of S-aryl dithiocarbamates was prepared through the reactions of classical organometallic reagents with tetramethyllitium disulfide (Jen and Cava, 1982; Knochel et al., 2006) (Scheme 1A). The reactions of sodium dialkyldithiocarbamates with diaryliodonium salts (Chen et al., 1987), aryl halide (Liu and Bao, 2007) or aryl boronic acid (Gao et al., 2018) were also proved to be an effective strategy (Scheme 1B). Recently, the three-component reactions of amines, carbon disulfide, and diverse electrophiles including alkyl halides (Azizi et al., 2006), aryl halides (Bhadra et al., 2008), aryldiazonium fluoroborates (Chatterjee et al., 2011), pentafluorobenzonitrile (Yin et al., 2015), and phenylboronic acid (Qi et al., 2016) (Scheme 1C) have been achieved by some research groups. Moreover, the cross-coupling reactions of tetraalkylthiuram disulfide with aryl iodide (Dong et al., 2017; Cao et al., 2018; Wu and Yan, 2019), phenylboronic acid (Xu et al., 2018), diaryl disulfides (Peng et al., 2019), or diaryliodonium salts (Zeng et al., 2017b) were also successively established by some chemists (Scheme 1D). However, these methods always suffer from one or more disadvantages such as toxic reagents, multiple reaction steps or flammable and explosive substrates, which limit their applications. To our knowledge, the synthesis of S-aryl dithiocarbamates using thiuram reagents (tetraalkylthiuram disulfides (TATD), or tetraalkylthiuram monosulfide (TATM)) and arylsilanes as the coupling partners has not been documented so far. As a continuation of our interest in the cross-coupling of tetraalkylthiuram disulfide (Wu et al., 2018; Lai et al., 2019a; Cheng et al., 2019; Hu et al., 2020), herein we wish to report the first example of copper-mediated C-S bond construction by cross-coupling of arylsilanes with thiuram reagents (TATD or TMTM) in the presence of CuF2 and N ligand (Scheme 1E), which would be an alternative way for the synthesis of S-aryl dithiocarbamates.

## Result and Discussion

Initially, the reaction parameters were optimized using trimethoxy (phenyl)silane (1a) and tetramethylthiuram disulfide (TMTD, 2a), and the results were summarized in Table 1. Firstly, the reaction of 1a (0.1 mmol) and 2a (0.2 mmol) was performed in the presence of CuF2 (3 equiv.) together with 20 mol% of CoCl2 in Toluene at 120°C. To our delight, the initial reaction conditions provided the desired product 3a (phenyl dimethylcarbamodithioate) in 22% yield (Table 1, entry 1). The exact structure of 3a was confirmed by NMR and HRMS spectra. When the reaction was carried out in the absence of CuF2, it didn’t produce any products (Table 1, entries 2 and 4). However, the reaction gave 23% yield of product 3a when CoCl2 was removed from the reaction system (Table 1, entry 3). The control experiment clearly indicated that CuF2 was indispensable for this reaction. Inspired by the reported literature (Clarke., 2005; McManus et al., 2006; Fihri et al., 2007; Hachiya et al., 2010; Wu et al., 2016; Sahani et al., 2018; Luo et al., 2020), some nitrogen and phosphorus ligands were screened (Table 1, entries 5-15, 0–78%), and 1, 10-phenanthroline was proved to be the optimized N ligand, affording the product 3a in 82% yield (Table 1, entry 6). Subsequently, other fluoride activators for the C-Si bond cleavage were evaluated in this reaction (Table 1, entries 16-17), but all of them turned out to be invalid. The effect of solvents such as Xylene, Mesitylene, 1,4-Dioxane, Acetonitrile, DMF and DMSO were also examined, and the experimental results showed that Toluene was the most suitable candidate with remarkably higher yields (Table 1, entry 6 vs entries 18-23, 82% vs 0–43%). Furthermore, the effect of CuF2 and N ligand loading was investigated (Table 1, entries 24-28, 26–74%). The obtained results revealed that a relatively lower reaction efficiency was detected in these reactions. Further optimization indicated that the temperature also played an important role in this transformation, and 80°C was identified as the ideal reaction temperature (Table 1, entry 6 and entries 29-31, 88% vs 59–85%). Meanwhile, the reaction time was also examined (Table 1, entries 32-33, 54–84%), and 16 h was found to be the best choice. Thus, the reaction efficiently proceeded when 3 equiv. of CuF2 was used in combination with 1,10-phenanthroline (2 equiv.) in Toluene at 80°C for 16 h. Noteworthily, the combination CuF2/phenantroline acted as the activator of C-Si bond, and also acted as the promoter on the formation of C-S bond.

**TABLE 1:** Optimization of reaction conditions ^a^.

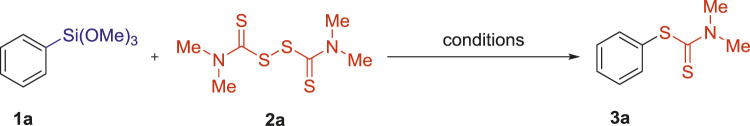					
Entry	Promoter	Ligand (Equiv.)	Solvent	T (°C)	Yields of 3a (%)^b^
1^c^	CuF2	-	Toluene	120	22
2	-	-	Toluene	120	0
3	CuF2	-	Toluene	120	23
4	CoCl2	-	Toluene	120	0
5	CuF2	bipyridine (2)	Toluene	120	61
6	CuF2	1,10-phenanthroline (2)	Toluene	120	82
7	CuF2	pyridine (2)	Toluene	120	78
8	CuF2	N,N,N′,N′-tetramethylethylenediamine (2)	Toluene	120	0
9	CuF2	2,2’:6′,2’’-terpyridine (2)	Toluene	120	43
10	CuF2	(R,R)-2,2’-(2,6-pyridinediyl)bis (4-isopropyl-2-oxazoline (2)	Toluene	120	39
11	CuF2	8-benzoylaminoquinoline (2)	Toluene	120	76
12	CuF2	1,2-bis(diphenylphosphino)ethane (2)	Toluene	120	<5
13	CuF2	2,2′-bis(diphenylphosphino)-1,1′-biphenyl (2)	Toluene	120	<5
14	CuF2	1,1′-bis(diphenylphosphino)ferrocene (2)	Toluene	120	<5
15	CuF2	(R)-(+)-2,2′-bis(diphenylphosphino)-1,1′-binaphthyl (2)	Toluene	120	11
16	AgF	1,10-phenanthroline (2)	Toluene	120	0
17	CsF	1,10-phenanthroline (2)	Toluene	120	0
18	CuF2	1,10-phenanthroline (2)	Xylene	120	38
19	CuF2	1,10-phenanthroline (2)	Msitylene	120	43
20	CuF2	1,10-phenanthroline (2)	1,4-Dioxane	120	20
21	CuF2	1,10-phenanthroline (2)	Acetonitrile	120	42
22	CuF2	1,10-phenanthroline (2)	DMF	120	0
23	CuF2	1,10-phenanthroline (2)	DMSO	120	0
24	CuF2	1,10-phenanthroline (3)	Toluene	120	58
25	CuF2	1,10-phenanthroline (0.5)	Toluene	120	74
26	CuF2	1,10-phenanthroline (2)	Toluene	120	37
27^d^	CuF2	1,10-phenanthroline (2)	Toluene	120	55
28^e^	CuF2	1,10-phenanthroline (2)	Toluene	120	26
29	CuF2	1,10-phenanthroline (2)	Toluene	100	85
30	CuF2	1,10-phenanthroline (2)	Toluene	80	88
31	CuF2	1,10-phenanthroline (2)	Toluene	60	59
32^f^	CuF2	1,10-phenanthroline (2)	Toluene	120	84
33^g^	CuF2	1,10-phenanthroline (2)	Toluene	120	54

a Trimethoxy (phenyl)silane 1a (0.10 mmol), Tetramethylthiuram disulfide 2a (0.20 mmol), promoter (3.0 equiv.), and Toluene (1 ml) for 16 h, under air.

b Isolated yields.

c 20 mol% of CoCl2 was added.

d Promoter (4.0 equiv.).

e Promoter (2.0 equiv.).

f 24 h.

g 48 h.

Having the optimized conditions in hand, we then proceeded to explore the scope of the reaction with respect to both the organosilane reagents and the thiuram disulfides (Table 2 and Table 3). Generally, phenylsilanes bearing diverse substituents such as methyl, methoxyl, tert-butyl, chloro and fluoro groups offered the desired products in moderate to good yields. Notably, this reaction tolerated the electron-rich arylsilanes (Table 2, 3b-g), as (4-methylphenyl) trimethoxylsilane, (4-methoxyphenyl) trimethoxylsilane and (4-(tert-butyl)phenyl) trimethoxylsilane coupled efficiently with tetramethylthiuram disulfide 2a to give 3b-d in 65–72% yields. When methyl, methoxyl, were introduced into the meta position of phenylsiloxanes, slight lower yields were obtained (3e-g, 43–55%), which may be caused by steric hindrance effect. Excellent yields were got for electron-deficient arylsilanes such as (4-chlorophenyl)trimethoxysilane and (4-fluorophenyl) trimethoxysilane. Compared with electron-rich substituents, arylsilanes with electron-withdrawing groups on the aromatic ring presented relatively higher reactivity (3b-c vs 3h-i, 65–72% vs 78–93%). This result makes the said cross-coupling reaction particularly attractive for further transformation by transition-metal-catalyzed coupling reactions. Pleasingly, these reaction conditions were also compatible with trimethoxy (4-vinylphenyl)silane, 1-(trimethoxysilyl)naphthalene and 2-furan-trimethoxysilane, which provided the corresponding products 3l-n in 52–72 yields.

**TABLE 2:** Reactions of arylsilanes 1) with tetramethylthiuram disulfide (2a) ^a^ ^,^ ^b^.

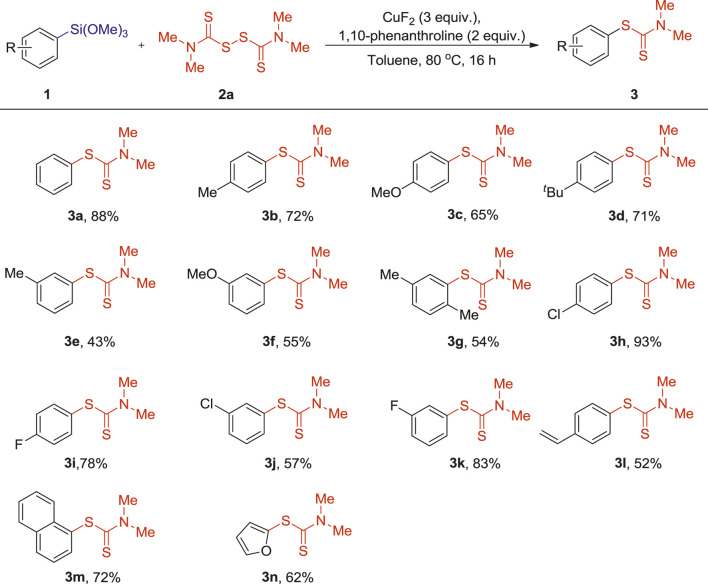

a 1 (0.1 mmol), 2a (0.2 mmol), CuF2 (3 equiv.), 1,10-phenanthroline (2 equiv.), Toluene (1 ml), 80°C, 16 h, under air.

b Isolated yields.

**TABLE 3:** Reactions of arylsilanes 1) with tetraalkylthiuram disulfides (2) ^a^ ^,^ ^b^.

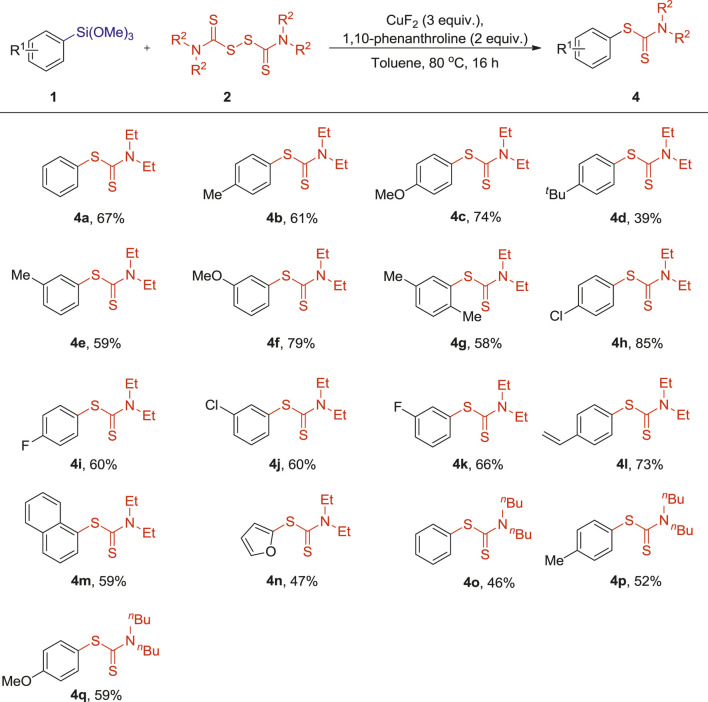

a 1 (0.10 mmol), 2 (0.20 mmol), CuF2 (3 equiv.), 1,10-phenanthroline (2 equiv.), Toluene (1 ml), 80°C, 16 h.

b Isolated yields.

This cross-coupling reaction also demonstrated a good tolerance toward other N,N,N′,N′-tetraalkylthiuram disulfides as shown in Table 2. The N,N,N′,N′-tetraethylthiuram disulfide (TETD, 2b) showed a good reactivity and furnished the corresponding S-aryl dithiocarbamates products in moderate to good yields (4a-n, 39–85%). Comparatively, the reaction of N,N,N′,N′-tetrabutylthiuram (TBTD, 2c) and arylsilanes showed relatively lower reactivity, and afforded lower yields of the corresponding products (4o-q, 46–59%). It is worth noting that the yields of the resulting products were modulated by the presence of different alkyl substituents on the tetraalkylthiuram disulfides. Slightly lower yields were obtained when longer chain-substituted tetraalkylthiuram disulfides were used in these reactions (3a vs 4a and 4°).

**SCHEME 2:**
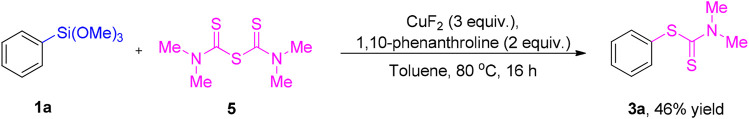
Initial cross-coupling reaction of trimethoxy (phenyl)silane and TMTM ^a,b^. a Reaction conditions: 1 (0.10 mmol), 5 (0.20 mmol), CuF2 (3 equiv.), 1,10-phenanthroline (2 equiv.), Toluene (1 ml), 80°C, 16 h b Isolated yields.

To further evaluate the applicability of this reaction, the reactivity of trimethoxy (phenyl)silane (1a) was investigated using tetramethylthiuram monosulfide (TMTM, 5) as the coupling partner (Wu et al., 2020). As expected, the cross-coupling reaction occurred smoothly, and the phenyl dimethylcarbamodithioate 3a was formed in 46% yield (Scheme 2).

In order to find the appropriate conditions to achieve an ideal yield, we spent a bit more time on the optimization of reaction conditons. Some bidentate, tridentate N ligands as well as diphoshines ligands and their loading to this reaction were tested, and a summative result of the optimization was presented in Supplementary Table S1. After the simple optimization, we found that 1 equiv. of 2, 2′-bipyridine increased the yield to 68% (Supplementary Table S1, entry 3), N,N,N′,N′-tetramethylethylenediamine, 2,2’:6′,2’’-terpyridine; (R,R)-2,2’-(2,6-pyridinediyl)bis (4-isopropyl-2-oxazoline, 8-benzoylaminoquinoline, 1,2-bis(diphenylphosphino)ethane, 2,2′-bis(diphenylphosphino)-1,1′-biphenyl, 1,1′-bis(diphenylphosphino)ferrocene and (R)-(+)-2,2′-bis(diphenylphosphino)-1,1′-binaphthyl resulted in a relatively lower yields (Supplementary Table S1, entries 4-11). It probably because of the coordination of 2, 2′-bipyridine with copper, which provided a more stable and active copper intermediate for the said cross-coupling reaction. After the simple optimization, we found that 1 equiv. of 2, 2′-bipyridine acted as the suitable N ligand. With the new optimized reaction conditions in hand, some more substituted arylsilanes were subjected to this reaction and the results were summarized in Table 4.

**TABLE 4:** Reactions of arylsilanes 1) with tetramethylthiuram monosulfide (5) ^a^ ^,^ ^b^.

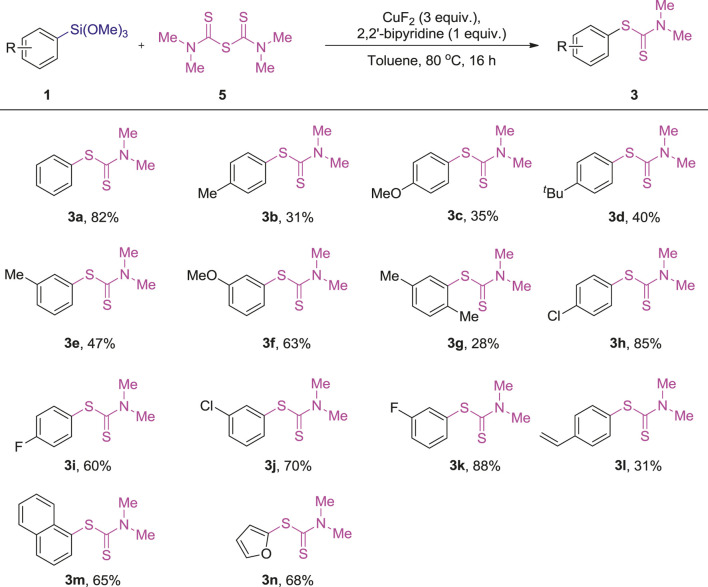

a 1 (0.10 mmol), 5 (0.20 mmol), CuF2 (3 equiv.), 2, 2′-bipyridine (1 equiv.), Toluene (1 ml), 80°C, 16 h.

b Isolated yields.

In general, the results obtained from the cross-coupling reaction of arylsilanes 1) with tetramethylthiuram monosulfide (TMTM, 5) are different from the reaction with tetramethylthiuram disulfide (TMTD, 2a), in which the electron-rich arylsilanes are less active (3b-g, 28–63%). With regard to the electron-deficient arylsilanes, they showed a similar efficiency as the reaction with tetramethylthiuram disulfide (TMTD, 2a), and the products (3h-k) were provided in 60–88% yields. The 1-(trimethoxysilyl)naphthalene and 2-furan-trimethoxysilane also participated in this reaction to give the corresponding products (3m-n) in 65–68% yields, which are nearly the same results compared with the reaction with TMTD (2a). In contrast, the trimethoxy (4-vinylphenyl)silane exhibited a less activity in this reaction and displayed lower yield (3l, 31%).

**SCHEME 3:**
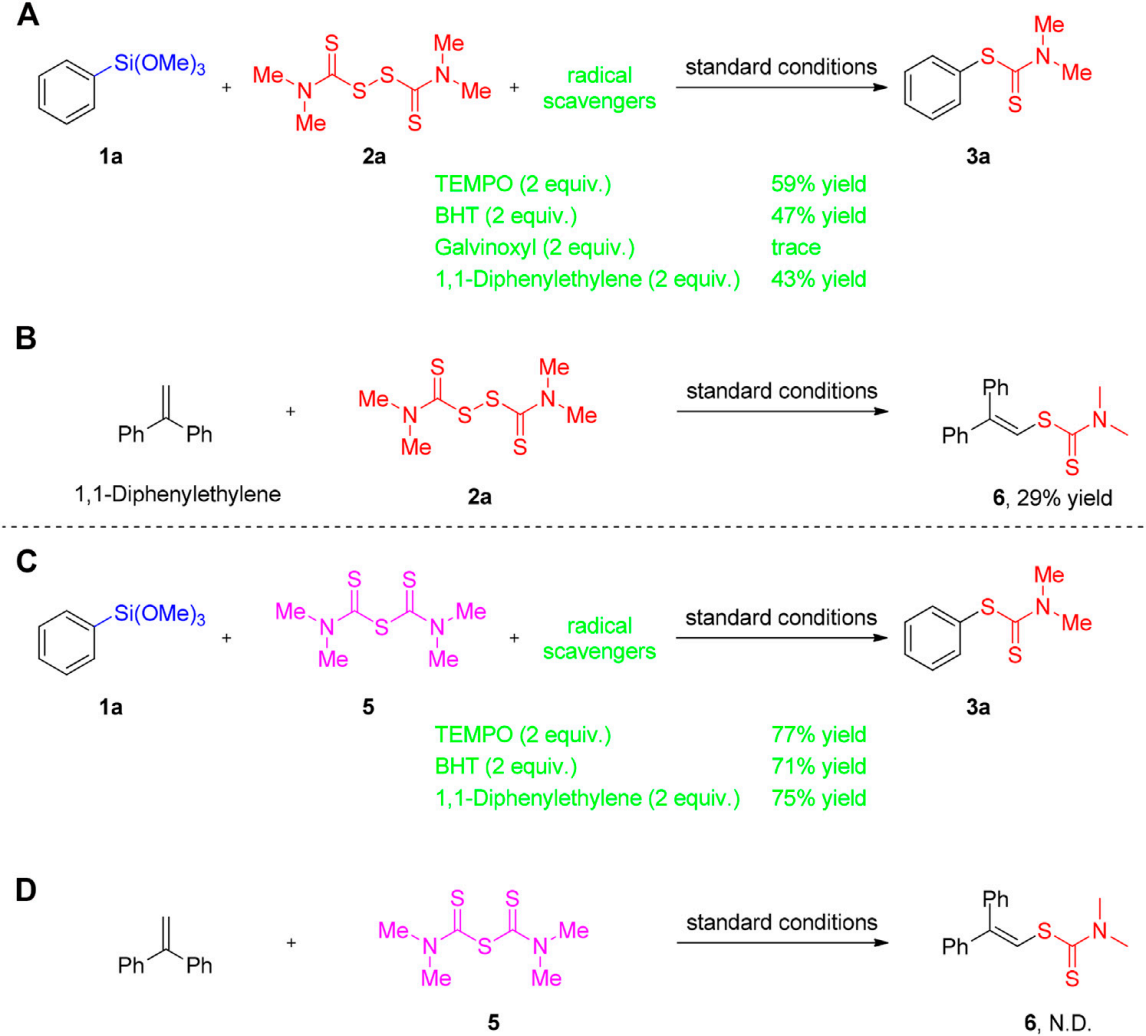
Mechanistic experiments.

In order to ascertain the mechanism, some control experiments were conducted and the results were exhibited in Scheme 3. When 2 equiv. of radical scavenger 2,2,6,6-tetramethyl-1-piperidinyloxyl (TEMPO), butylated hydroxyl toluene (BHT), galvinoxyl free radical or 1,1-diphenylethylene were added to the reaction of 1a and 2a under the standard conditions, a substantial decrease of the reaction efficiency was observed (Scheme 3A). Subsequently, the radical quencher 1,1-diphenylethylene was added to the tetraalkylthiuram disulfides participated reaction system, the thiuram radical was captured to give the corresponding product six in 29% yield (Scheme 3B). The above mentioned results illustrating that a radical process may be exist in the reaction of 1a and 2a. In sharp contrast, when the reactions were occurred between 1a and 5 in the presence of radical inhibitors (2.0 equiv of TEMPO, BHT) or 1,1-diphenylethylene, which gave the desired product 3a in 77, 71, and 75% yields, respectively (Scheme 3C). Furthermore, no desired product six was observed when 1,1-diphenylethylene react with 5 (TMTM) under the standard conditions (Scheme 3D). These results suggesting that the reaction of 1a and 5 is more likely to be an ionic-type pathway.

**SCHEME 4:**
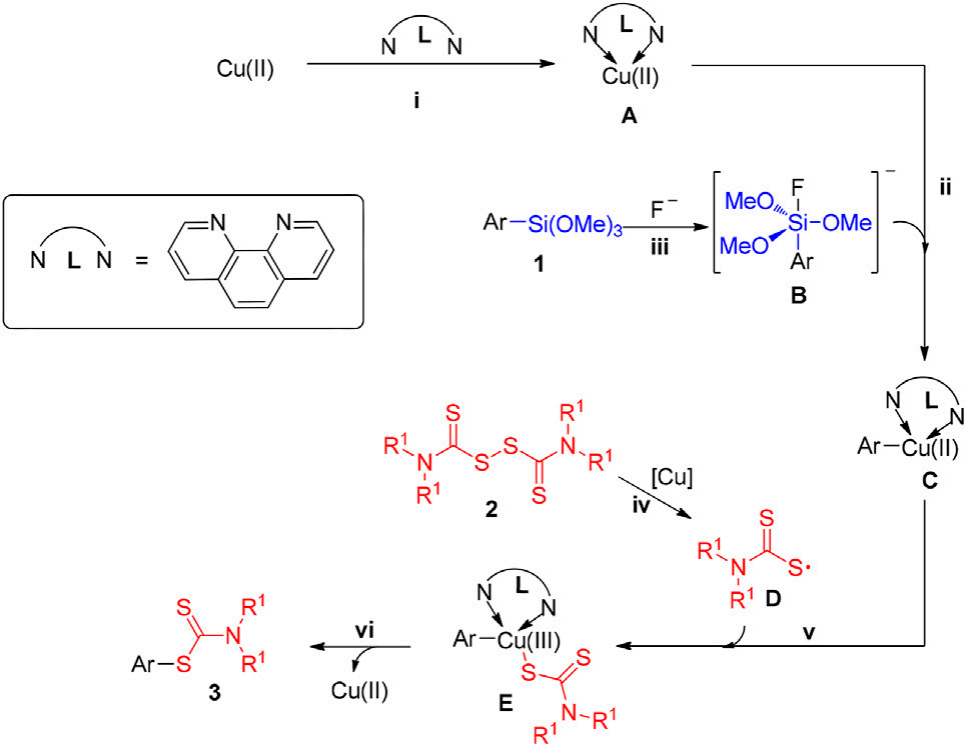
Proposed reaction mechanism for the reaction of arylsilanes with TMTD.

Considering the experimental evidence as well the previous reports (Lu et al., 2020; Dong et al., 2017; Luo et al., 2020; Hao et al., 2020; Lai et al., 2019b), a plausible reaction mechanism was tentatively proposed and described in Scheme 4. Firstly, the coordination of 1, 10-phenanthroline with copper salts to produce the copper complex A. Simultaneously, the C-Si cleavage process occurred lead to the intermediate B, which activated by fluoride ion (Sugiyama et al., 2008). In step ii, the reaction of intermediate B with copper complex A generates the Cu(II) complex C. Subsequently, thiuram radical D may be formed through the homolysis of tetramethylthiuram disulfide at 80°C probably assisted by Cu(II). Then, the interreaction of Cu(II) complex C with thiuram radical D to provide the intermediate E, which undergoes reductive elimination to yield the desired product three along with the release of Cu(II) species.

**SCHEME 5:**
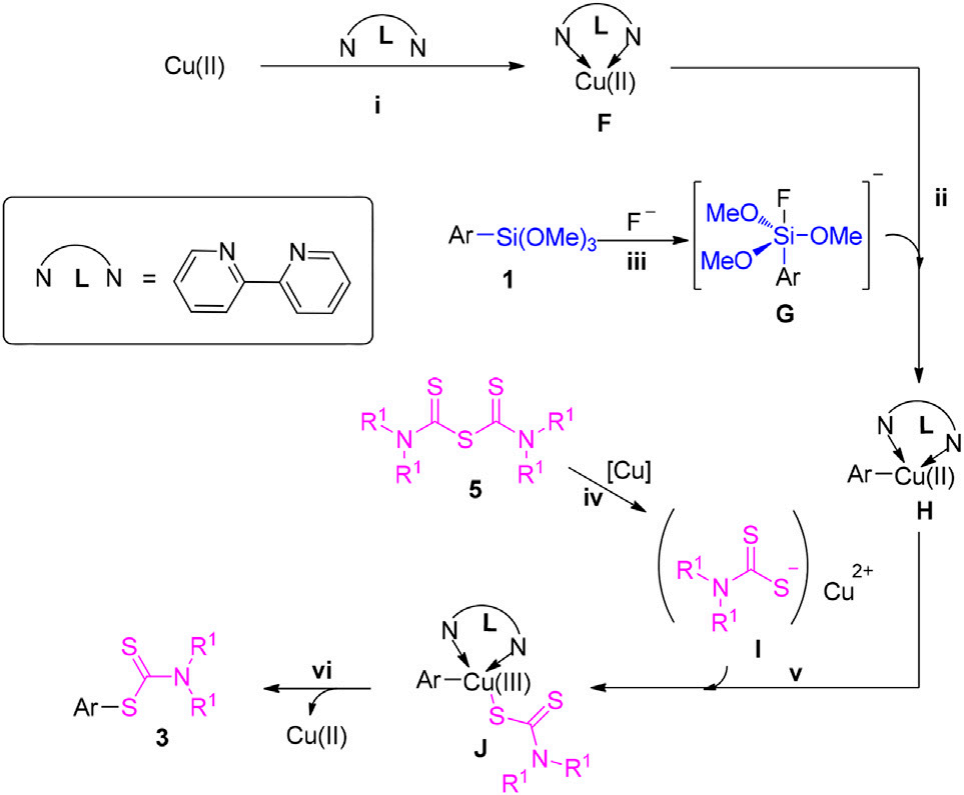
Proposed reaction mechanism for the reaction of arylsilanes with TMTM.

With regard to the reaction pathway between 1a and 5 (TMTM), a plausible ionic-type reaction mechanism was tentatively proposed according to the obtained results as well as the reported literatures (Dong et al., 2017; Luo et al., 2020; Wu et al., 2020) and described in Scheme 5. Analogously, the initial coordination of bipyridine with copper salts to produce the copper complex F. Concurrently, the intermediate G is generated by the C-Si cleavage manner, which activated by fluoride ion (Sugiyama et al., 2008). Then, the intereaction of intermediate G with copper complex F to generate the Cu(II) complex H. In the meantime, nucleophile F probably produces by the intereaction of copper ion with 5 at 80°C. Subsequently, the interreaction of Cu(II) complex H with nucleophile I to provide the intermediate J, which undergoes reductive elimination lead to the desired product three along with the release of Cu(II) species.

## Conclusion

In summary, we have developed an interesting methodology on the copper-promoted cross-coupling of arylsilanes and thiuram reagents (TATD or TMTM), affording the valuable S-aryl dithiocarbamates in moderate to good yields. This facile strategy allows practical and friendly reaction conditions, which significantly broadens the substrate scope, improves the functional group compatibility, and emphasizes the synthetic application in complex molecules. It offers not only a protocol for the streamlined synthesis of S-aryl dithiocarbamates from cheap and stable substrates, but also a new example for the application of Hiyama cross-coupling in biological interesting molecules’ construction.

## Data Availability

The original contributions presented in the study are included in the article/Supplementary Material, further inquiries can be directed to the corresponding authors.
