# Supplementary material for: Copper-Promoted Hiyama Cross-Coupling of Arylsilanes With Thiuram Reagents: A Facile Synthesis of Aryl Dithiocarbamates
Source: Front Chem. 2022 Apr 26;10:867806. doi: 10.3389/fchem.2022.867806 (PMC9087285; doi:10.3389/fchem.2022.867806)
Supplement: Supplementary file 1 [file DataSheet1.pdf]

Supplementary Material

Copper-promoted Hiyama Cross-Coupling of Arylsilanes with Thiuram Reagents: A Facile Synthesis of Aryl Dithiocarbamates

Yiying Wang[1], Hongtao Shen[2], Jianhua Qiu[2], Mengqi Chen[2]*, Weimin Song[2]*, Mingqin Zhao[1], Longfei Wang[1], Feng Bai[2], Hongxia Wang[2], Zhiyong Wu[1]*

[1] *Flavors and Fragrance Engineering & Technology Research Center of Henan Province, College of Tobacco Science, Henan Agricultural University, Zhengzhou 450002, P. R. China*

[2] *Technology Center, China Tobacco Henan Industrial Co., Ltd., Zhengzhou, 450000, P. R. China*

479820476@qq.com

gongyishi@126.com

zhiyongwu@henau.edu.cn

Contents

1. **General Information**……………………………..............……………………………...**S-2**
2. **Table S1.**………………………...………………………**S-2**
3. **Experimental Section**…………………………………...........………………………**.S-2**
4. **Characterization Data**…………………………………………...............………………**S-4**
5. **References**…………………………………………………...............………………**S-12**
6. **Copy of ^{1}H and ^{13}C NMR Spectra**……………………………..............………………**.S-13**

1 General information

All the reagents were obtained commercially and used without further purification. Silica gel was purchased from Qing Dao Hai Yang Chemical Industry Co. Analytical thin layer chromatography (TLC) was performed on precoated silica gel F_{254} plates. Compounds were visualized by irradiation with UV light (254 nm).

Analytical information: ^{1}H NMR and ^{13}C NMR spectra data were recorded by a BRUKER AVANCE III 400 MHz spectrometer (^{1}H 400 MHz, ^{13}C 100 MHz), using $CDCl_3$ as the solvent with tetramethylsilane (TMS) as the internal standard at room temperature. ^{1}H NMR spectral data are given as chemical shifts in ppm: followed by multiplicity (s-singlet; d-doublet; t-triplet; q-quartet; m-multiplet), number of protons and coupling constants. ^{13}C NMR chemical shifts are expressed in ppm. HRMS data were obtained using AB SCIEX Triple TOF 5600+ high resolution mass spectrometer (USA). The products listed below were determined by ^{1}H and ^{13}C NMR spectra. Infrared spectra were recorded with a Thermo Scientific Nicolet 6700 FT-IR Spectrometer. Melting points were determined using melting point X-4 (Gongyi Kerui) apparatus.

2 Table S1. Optimization of reaction conditions.[a]

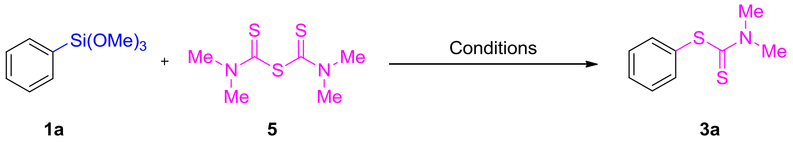

Entry	Catalyst	Ligand (equiv.)	Yield (%)[b]
1	CuF_2	1,10-phenanthroline (2)	46
2	CuF_2	pyridine (2)	37
3	CuF_2	2,2'-bipyridine (2)	68
4	CuF_2	*N*,*N*,*N'*,*N'*-tetramethylethylenediamine (2)	0
5	CuF_2	2,2':6',2''-terpyridine (2)	49
6	CuF_2	(*R*,*R*)-2,2'-(2,6-pyridinediyl)bis(4-isopropyl-2-oxazoline (2)	51
7	CuF_2	8-benzoylaminoquinoline (2)	55
8	CuF_2	1,2-bis(diphenylphosphino)ethane (2)	55

9	CuF_2	2,2'-bis(diphenylphosphino)-1,1'-biphenyl (2)	40
10	CuF_2	1,1'-bis(diphenylphosphino)ferrocene (2)	63
11	CuF_2	(*R*)-(+)-2,2'-bis(diphenylphosphino)-1,1'-binaphthyl (2)	57
**12**	**CuF_2**	**2,2'-bipyridine (1)**	**82**
13	CuF_2	2,2'-bipyridine (0.5)	79

[a] Reaction conditions: **1a** (0.10 mmol), **5** (0.20 mmol), CuF_2 (3.0 equiv.), ligand (0.5-2 equiv.), Toluene (1 mL), 80 °C, 16 h, under air.
[b] Isolated yields.

3 Experimental Section

(1) General procedures for the Synthesis of various arylsiloxanes

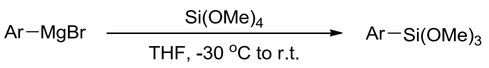

To a solution of tetramethyl orthosilicate (60 mmol) in 20 mL anhydrous THF was charged into a 100 mL round-bottom flask. The silane solution was then cooled to -30 °C, and the arylmagnesiumhalide solution was added dropwise (one drop per second). The solution was allowed to stir at -30 °C for 1 h and then at room temperature for 12 h. After the reaction finished, the mixture was then poured into 30 mL of hexane, and stirred for some time. Then the solution was washed with 3 × 25 mL of water, dried over Na_2SO_4, and concentrated in *vacuo*. Purification of the residue by short-path distillation to yield the desired arylsilanes.[1-2].

(2) General procedures for the reaction of arylsiloxanes with tetraalkhylthiuram disulfides

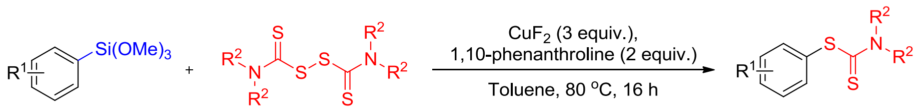

To a 10 mL tube, arylsiloxanes **1** (0.1 mmol) and tetraalkylthiuram disulfides **2** (0.2 mmol), CuF_2 (0.3 mmol), 1,10-phenanthroline (0.2 mmol) and toluene (1.0 mL) were added under air atmosphere. The resulting mixture was heated in an 80 °C oil bath with vigorous stirring for 16 h. Then, the reaction mixture was cooled to room temperature, quenched with a sat. NH_4Cl solution and subsequently extracted with ethyl acetate. The combined organic layers were dried over anhydrous Na_2SO_4, filtered and the solvent was evaporated under *vacuum*. The residue was purified by flash

chromatography using petroleum ether /ethyl acetate (10:1) as eluent to affording **3** or **4** in 39-93% yield. In general, the identity and purity of the products were confirmed by ^{1}H and ^{13}C NMR spectroscopy, HRMS and IR.

(3) General procedures for the reaction of arylsiloxanes with tetramethylthiuram monosulfide

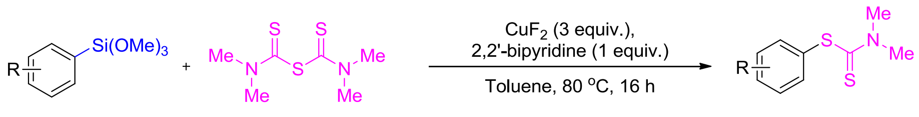

Under air atmosphere, arylsiloxanes **1** (0.1 mmol), tetramethylthiuram monosulfide **5** (0.2 mmol), CuF_2 (0.3 mmol), 2,2'-bipyridine (0.1 mmol) were charged into a 10 mL reaction tube, then Toluene (1 mL) was added into the tube. The resulting mixture was stirred at 80 °C for 16 h in oil bath, then cooled down to room temperature. The reaction mixture was quenched with a sat. NH_4Cl solution and subsequently extracted with ethyl acetate. The combined organic layers were dried over anhydrous $MgSO_4$, and the solvent was evaporated under *vacuum*. The crude product was purified by flash column chromatography on silica gel (elute: petroleum ether-EtOAc) yielding the products **3** in 28-88 yields. In general, the identity and purity of the products were confirmed by ^{1}H and ^{13}C NMR spectroscopy, HRMS and IR.

4 Characterization Data

phenyl dimethylcarbamodithioate (3a)

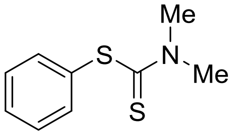

Purification by flash column chromatography on silica gel, R_f = 0.47; petroleum ether/EtOAc = 10/1; isolated yield = 88% (17.4 mg, with TMTD), 82% (16.1 mg, with TMTM); white solid; M.p. 87-89 °C; ^{1}H NMR (400 MHz, $CDCl_3$) ppm: 7.63-7.38 (m, 5H), 3.64-3.51 (s, 3H), 3.51-3.41 (s, 3H); ^{13}C NMR (100 MHz, $CDCl_3$) ppm: 197.6, 137.0, 131.7, 130.1, 129.1, 45.7, 42.0; IR (KBr) $_{vmax}$ 2922, 1497, 1472, 1439, 1376, 1253, 985, 966, 751, and 687 cm^{-1}; HRMS (EI) calcd. for $C_9H_{11}NS_2$: $[M]^+$: 197.0333, found: 197.0339.

4-methylphenyl diethylcarbamodithioate (3b)

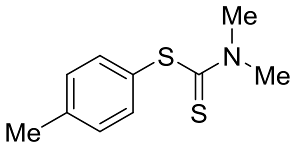

Purification by flash column chromatography on silica gel, R_f = 0.48; petroleum ether/EtOAc = 10/1; isolated yield = 72% (15.3 mg, with TMTD), 31% (6.5 mg, with TMTM); white solid; M.p. 108-109 ^{o}C; ^{1}H NMR (400 MHz, $CDCl_3$) ppm: 7.39-7.31 (m, 2H), 7.29-7.22 (m, 2H), 3.62-3.51 (s, 3H), 3.51-3.45 (s, 3H), 2.49-2.35 (s, 3H); ^{13}C NMR (100 MHz, $CDCl_3$) ppm: 198.2, 140.4, 136.8, 130.1, 128.3, 45.8, 42.0, 42.5, 21.5; IR (KBr) $_{vmax}$ 3384, 2974, 2925, 1489, 1450, 1407, 1372, 1247, 1149, 1089, 1050, 973, and 808 cm^{-1}; HRMS (EI) calcd. for $C_{10}H_{13}NS_2$: $[M]^+$: 211.0489, found: 211.0483.

4-methoxyphenyl dimethylcarbamodithioate (3c)

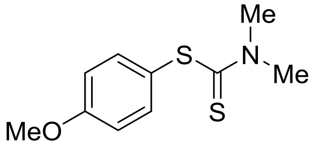

Purification by flash column chromatography on silica gel, R_f = 0.41; petroleum ether/EtOAc = 10:1; isolated yield = 65% (14.8 mg, with TMTD), 35% (7.9 mg, with TMTM); white solid; M.p. 85-86 ^{o}C; ^{1}H NMR (400 MHz, $CDCl_3$) ppm: 7.43-7.34 (m, 2H), 7.02-6.94 (m, 2H), 3.91-3.81 (s, 3H), 3.62-3.52 (s, 2H), 3.52-3.42 (s, 3H); ^{13}C NMR (100 MHz, $CDCl_3$) ppm: 198.8, 161.1, 138.5, 122.6, 114.7, 55.3, 45.8, 41.9; IR (KBr) ν_{max} 3422, 2923, 1589, 1571, 1493, 1433, 1403, 1375, 1248, 831, 634, and 648 cm^{-1}; HRMS (EI) calcd. for $C_{13}H_{12}N_2O_3$: $[M]^+$: 227.0439, found: 227.0431.

4-(*tert*-butyl)phenyl dimethylcarbamodithioate (3d)

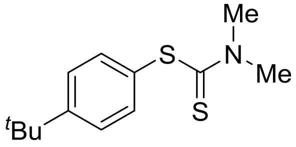

Purification by flash column chromatography on silica gel, R_f = 0.57; petroleum ether/EtOAc = 10/1; isolated yield = 71% (18.1 mg, with TMTD), 40% (10.2 mg, with TMTM); light yellow solid; M.p. 85-86 ^{o}C; ^{1}H NMR (400 MHz, $CDCl_3$) ppm: 7.50-7.43 (m, 2H), 7.43-7.36 (m, 2H), 3.62-3.53 (s, 3H), 3.53-3.46 (s, 3H), 1.49-1.32 (m, 9H); ^{13}C NMR (100 MHz, $CDCl_3$) ppm: 198.0, 153.2, 136.4, 128.2, 126.3, 45.7, 42.0, 34.8, 31.2, 30.0; IR (KBr) ν_{max} 3434, 2920, 2853, 1489, 1456, 1370, 1250, 1145, 1050, 983, 865, 812, and 702 cm^{-1}; HRMS (ESI) calcd. for $C_{13}H_{19}NS_2$: $[M+Na]^+$: 276.0851, found: 276.0857.

***m*-tolyl dimethylcarbamodithioate (3e)**[3]

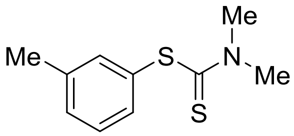

Purification by flash column chromatography on silica gel, R_f = 0.52; petroleum ether/ EtOAc = 10/1; isolated yield = 43% (9.1 mg, with TMTD), 47% (10.0 mg, with TMTM); deep yellow liquid; ^{1}H NMR (400 MHz, $CDCl_3$) ppm: 7.38-7.31 (m, 1H), 7.31-7.26 (m, 3H), 3.61-3.53 (s, 3H), 3.53-3.47 (s, 3H), 2.45-2.35 (s, 3H); ^{13}C NMR (100 MHz, $CDCl_3$) ppm: 197.9, 139.0, 137.4, 134.0, 131.4, 130.9, 128.9, 45.7, 42.0, 21.3; IR (KBr) ν_{max} 2956, 2901, 2865, 1488, 1374, 1361, 1249, 1148, 1114, 1013, 984, and 828 cm^{-1}.

3-methoxyphenyl dimethylcarbamodithioate (3f)[4]

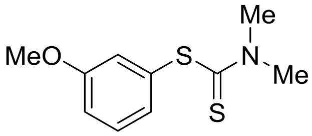

Purification by flash column chromatography on silica gel, R_f = 0.43; petroleum ether/EtOAc = 10/1; isolated yield = 55% (12.6 mg, with TMTD), 63% (14.4 mg, with TMTM); deep yellow liquid; ^{1}H NMR (400 MHz, $CDCl_3$) ppm: 7.38-7.32 (t, J = 7.9 Hz, 1H), 7.11-7.05 (m, 1H), 7.05-6.98 (m, 2H), 3.87-3.80 (s, 3H), 3.61-3.53 (s, 3H), 3.53-3.46 (m, 3H) ^{13}C NMR (100 MHz, $CDCl_3$) ppm: 197.4, 159.8, 132.5, 129.8, 129.1, 121.9, 116.3, 55.4, 45.7, 42.0; IR (KBr) ν_{max} 2957, 2917, 2850, 1590, 1574, 1472, 1276, 1227, 1042, 974, 859, 770, and 679 cm^{-1}.

2,5-dimethylphenyl dimethylcarbamodithioate (3g)[6]

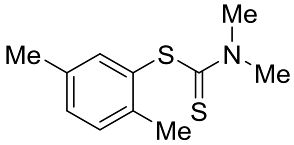

Purification by flash column chromatography on silica gel, R_f = 0.48; petroleum ether/EtOAc = 10/1; isolated yield = 54% (12.2 mg, with TMTD), 28% (6.4 mg, with TMTM); white solid; M.p. 56-57 ^{o}C ^{1}H NMR (400 MHz, $CDCl_3$) ppm: 8.43 (d, J = 5.3 Hz, 1H), 8.13 (dd, J = 8.4, 1.3 Hz, 2H), 7.66-7.58 (m, 2H), 7.48 (t, J = 6.9 Hz, 2H), 7.43 (dd, J = 5.3, 1.8 Hz, 1H), 5.47 (s, 2H); ^{13}C NMR (100 MHz, $CDCl_3$) ppm: 166.09, 157.60, 150.19, 133.68, 133.41, 129.85, 129.55, 128.54, 126.30, 124.95, 66.44; IR (KBr) ν_{max} 3434, 2920, 2853, 1489, 1456, 1370, 1250, 1145, 1050, 983, 865, 812, and 702 cm^{-1}.

4-chlorophenyl dimethylcarbamodithioate (3h)[3]

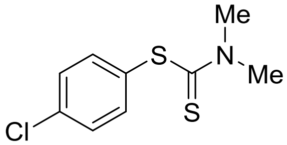

Purification by flash column chromatography on silica gel, R_f = 0.48; petroleum ether/ EtOAc = 10/1; isolated yield = 93% (21.5 mg, with TMTD), 85% (19.7 mg, with TMTM); white solid; M.p. 89-90 °C; ^{1}H NMR (400 MHz, $CDCl_3$) ppm: 7.45-7.35 (m, 4H), 3.64-3.51 (s, 3H), 3.51-3.41 (s, 3H); ^{13}C NMR (100 MHz, $CDCl_3$) ppm: 196.8, 138.2, 136.6, 130.2, 129.4, 45.8, 42.0; IR (KBr) ν_{max} 3422, 2925, 1504, 1471, 1376, 1251, 1152, 1083, 1015, 973, 814, and 749 cm^{-1}; HRMS (EI) calcd. for $C_{11}H_{16}ClNS_2$: $[M]^+$: 230.9943, found: 230.9936.

4-fluorophenyl dimethylcarbamodithioate (3i)[3]

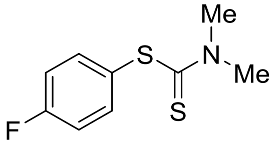

Purification by flash column chromatography on silica gel, R_f = 0.37; petroleum ether/EtOAc = 10/1); isolated yield = 78% (16.9 mg, with TMTD), 60% (13.0 mg, with TMTM); light yellow solid; M.p. 79-80 °C; ^{1}H NMR (400 MHz, $CDCl_3$) ppm: 7.48-7.40 (m, 2H), 7.17-7.09 (m, 2H), 3.59-3.51 (s, 3H), 3.51-3.46 (s, 3H); ^{13}C NMR (100 MHz, $CDCl_3$) ppm: 197.5, 165.2, 162.7, 139.1 (139.2, 139.1, d, J = 8.7 Hz), 127.2 (127.3, 127.1, d, J = 3.7 Hz), 116.4 (116.5, 116.3, d, J = 22 Hz), 45.8, 41.9; IR (KBr) ν_{max} 3438, 2926, 1586, 1488, 1377, 1252, 1217, 1157, 1090, 1015, 980, 937, 831, and 815 cm^{-1}.

3-chlorophenyl dimethylcarbamodithioate (3j)[3]

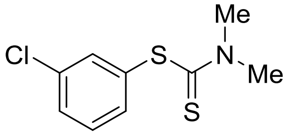

Purification by flash column chromatography on silica gel, R_f = 0.32; petroleum ether/ EtOAc = 10/1; isolated yield = 57% (13.3 mg, with TMTD), 70% (16.3 mg, with TMTM); deep yellow liquid; ^{1}H NMR (400 MHz, $CDCl_3$) ppm: 7.49-7.46 (m, 1H), 7.46-7.42 (m, 1H), 7.39-7.35 (m, 2H), 3.60-3.53 (s, 3H), 3.53-3.46 (s, 3H); ^{13}C NMR (100 MHz, $CDCl_3$) ppm: 196.4, 136.7, 135.2, 134.4, 133.3, 130.2, 130.1, 45.7, 42.1; IR (KBr) ν_{max} 3428, 2923, 2852, 1628, 1500, 1399, 1271, 1244, 1151, 1003, 964, 8523, and 720 cm^{-1}.

3-fluorophenyl dimethylcarbamodithioate (3k)[4]

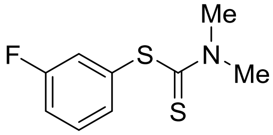

Purification by flash column chromatography on silica gel, R_f = 0.37; petroleum ether/ EtOAc = 10/1; isolated yield = 83% (17.8 mg, with TMTD), 88% (19.0 mg, with TMTM); light yellow solid; M.p. 54-55 °C; ^{1}H NMR (400 MHz, $CDCl_3$) ppm: 7.45-7.37 (m, 1H), 7.29-7.24 (m, 1H), 7.24-7.14 (m,

2H), 3.56-3.51 (s, 3H), 3.51-3.46 (s, 3H); ^{13}C NMR (100 MHz, $CDCl_3$) ppm: 196.5, 163.7, 161.2, 133.3 (133.4, 133.3, d, *J* = 8.1 Hz), 132.7 (132.8, 132.7, d, *J* = 3.3 Hz), 130.2 (130.3, 130.2, d, *J* = 8.12 Hz), 123.9 (124.0 123.8, d, *J* = 22 Hz), 117.3 (117.4, 117.2, d, *J* = 17.2 Hz), 45.7, 42.1; IR (KBr) ν_{max} 3435, 2926, 1581, 1473, 1375, 1250, 1217, 1145, 1052, 985, 880, and 781 cm^{-1}.

4-vinylphenyl dimethylcarbamodithioate (3l)

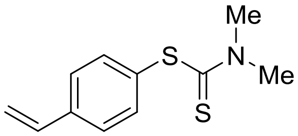

Purification by flash column chromatography on silica gel, R_f = 0.45; petroleum ether/ EtOAc = 10/1; isolated yield = 52% (11.6 mg, with TMTD), 31% (6.9 mg, with TMTM); light yellow solid; M.p. 66-67 ^{o}C; ^{1}H NMR (400 MHz, $CDCl_3$) ppm: 7.53-7.45 (m, 2H), 6.80-6.69 (m, 1H), 5.88-5.78 (m, 1H), 5.39-5.31 (m, 1H), 3.61-3.51 (s, 3H), 3.51-3.45 (s, 3H); ^{13}C NMR (100 MHz, $CDCl_3$) ppm: 197.6, 139.2, 137.1, 136.1, 130.7, 126.9, 115.6, 45.7, 42.0; IR (KBr) ν_{max} 2923, 2852, 1486, 1375, 1248, 1146, 1108, 1013, 976, 906, and 836 cm^{-1}; HRMS (EI) calcd. for $C_{11}H_{13}NS_2$: $[M]^+$: 223.0489; found: 223.0485.

naphthalen-1-yl dimethylcarbamodithioate (3m)

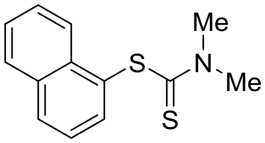

Purification by flash column chromatography on silica gel, R_f = 0.40; petroleum ether/ EtOAc = 10/1; isolated yield = 72% (17.9 mg, with TMTD), 65% (16.2 mg, with TMTM); yellow solid; M.p. 146-147 °C; ^{1}H NMR (400 MHz, $CDCl_3$) ppm: 8.29-8.22 (m, 1H), 8.04-7.97 (d, *J* = 8.2 Hz, 1H), 7.93-7.87 (m, 1H), 7.78-7.72 (m, 1H), 7.59-7.48 (m, 3H), 3.71-3.61 (m, 3H), 3.61-3.52 (s, 3H); ^{13}C NMR (100 MHz, $CDCl_3$) ppm: 196.6, 137.1, 135.1, 134.2, 131.5, 129.0, 128.7, 127.2, 126.3, 125.8, 45.6, 42.2; IR (KBr) ν_{max} 2922, 1500, 1376, 1251, 1146, 987, 967, 909, 860, 796, 771, and 740 cm^{-1}; HRMS (ESI) calcd. for $C_{13}H_{13}NS_2$: $[M+Na]^{+}$: 270.0382, found: 270.0382.

furan-2-yl dimethylcarbamodithioate (3n)

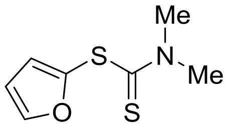

Purification by flash column chromatography on silica gel, R_f = 0.40; petroleum ether/ EtOAc = 10/1; isolated yield = 62% (11.7 mg, with TMTD), 68% (12.7 mg, with TMTM); white solid; M.p. 89-90 °C; ^{1}H NMR (400 MHz, $CDCl_3$) ppm: 7.67-7.61 (dd, *J* = 5.32 Hz, 1H), 5.26-5.22 (m, 1H), 7.18-

7.11 (m, 1H), 3.60-3.51 (s, 3H), 3.51-3.42 (s, 3H); ^{13}C NMR (100 MHz, $CDCl_3$) ppm: 197.4, 138.8, 133.5, 129.2, 127.9, 46.1, 41.8; IR (KBr) ν_{max} 3428, 2923, 1450, 1399, 1376, 1244, 1151, 1051, 1003, 964, 853, and 720 cm^{-1}.

phenyl diethylcarbamodithioate (4a)

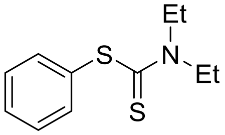

Purification by flash column chromatography on silica gel, R_f = 0.44; petroleum ether/ EtOAc = 15/1; isolated yield = 67% (15.1 mg, with TETD); pale yellow liquid; ^{1}H NMR (400 MHz, $CDCl_3$) ppm: 7.55-7.39 (m, 5H), 4.10-3.38 (q, *J* = 6.9, 3.9 Hz, 2H), 3.95-3.78 (q, *J* = 7.0 Hz, 2H), 1.45-1.35(t, *J* = 7.1 Hz, 3H), 1.35-1.26(m, 3H); ^{13}C NMR (100 MHz, $CDCl_3$) ppm: 137.2, 131.6, 130.0, 129.0, 49.9, 47.3, 12.8, 11.6; IR (KBr) ν_{max} 2969, 2928, 2849, 1487, 1438, 1411, 1267, 1205, 1141, and 747 cm^{-1}; HRMS (EI) calcd. for $C_{11}H_{15}NS_2$: $[M]^+$: 225.0646; found: 225.0641.

***p*-tolyl diethylcarbamodithioate (4b)**

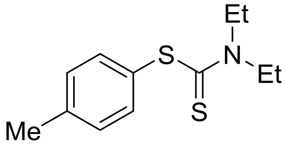

Purification by flash column chromatography on silica gel, R_f = 0.43; petroleum ether/EtOAc = 15/1; isolated yield = 61% (14.5 mg, with TETD); yellow solid; M.p. 75-76 °C; ^{1}H NMR (400 MHz, $CDCl_3$) ppm: 7.41-7.33 (d, *J* = 8.0 Hz, 2H), 7.31-7.22 (d, *J* = 7.6 Hz, 2H), 4.10-3.96 (q, *J* = 6.9 Hz, 2H), 3.94-3.40 (q, *J* = 6.9 Hz, 3H), 2.45-2.36 (s, 3H), 1.45-1.36 (t, *J* = 7.0 Hz, 3H), 1.34-1.22 (t, *J* = 7.0 Hz, 3H); ^{13}C NMR (100 MHz, $CDCl_3$) ppm: 196.5, 140.3, 137.0, 130.0, 128.2, 50.0, 47.2, 21.5, 12.7, 11.6; IR (KBr) ν_{max} 2977, 2930, 1483, 1418, 1376, 1267, 1202, 1142, 1008, 978, 915, and 808 cm^{-1}; HRMS (ESI) calcd. for $C_{12}H_{17}NS_2$: $[M+Na]^+$: 262.0695; found: 262.0688.

4-methoxyphenyl diethylcarbamodithioate (4c)

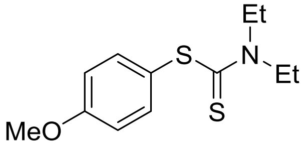

Purification by flash column chromatography on silica gel, R_f = 0.48; petroleum ether/EtOAc = 15/1; isolated yield = 74% (18.9 mg, with TETD); white solid; M.p. 73-74 °C; ^{1}H NMR (400 MHz, $CDCl_3$) ppm: 7.43-7.35 (m, 2H), 7.01-6.93 (m, 2H), 4.11-3.97 (q, *J* = 7.0 Hz, 3H), 3.91-3.81 (m, 5H), 1.45-1.36 (t, *J* = 7.0 Hz, 3H), 1.34-1.25 (t, *J* = 7.0 Hz, 3H); ^{13}C NMR (100 MHz, $CDCl_3$) ppm: 197.2, 161.1, 138.6, 122.4, 114.7, 55.3, 50.0, 47.1, 12.7, 11.6; IR (KBr) ν_{max} : 2957, 2931, 1589, 1491, 1440,

1416, 1293, 1268, 1248, 1185, 1174, 1145, 1028, and 827 cm^{-1}; HRMS (EI) calcd. for $C_{16}H_{17}NOS_2$: $[M]^+$: 255.0752, found: 255.0742.

4-(*tert*-butyl)phenyl diethylcarbamodithioate (4d)

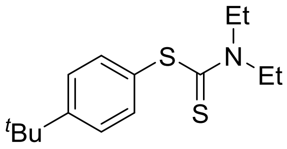

Purification by flash column chromatography on silica gel, R_f = 0.43; petroleum ether/EtOAc = 15/1; isolated yield = 39% (10.9 mg, with TETD); yellow solid; M.p. 106-107 °C; ^{1}H NMR (400 MHz, $CDCl_3$) ppm: 7.49-7.43 (m, 2H), 7.43-7.37 (m, 2H), 4.09-3.98 (q, *J* = 6.8 Hz, 2H), 3.93-3.81 (q, *J* = 7.0 Hz, 2H), 1.47-1.37 (t, *J* = 7.0 Hz, 3H), 1.37-1.31 (s, 9H), 1.31-1.27 (m, 3H); ^{13}C NMR (100 MHz, $CDCl_3$) ppm: 196.4, 153.1, 136.7, 128.1, 126.2, 49.8, 47.2, 34.8, 31.2, 12.8, 11.6; IR (KBr) ν_{max} 2977, 2962, 2932, 1459, 1485, 1439, 1417, 1268, 1207, 1142, 1091, 1067, 982, and 828 cm^{-1}; HRMS (ESI) calcd. for $C_{15}H_{23}NS_2$: $[M+Na]^+$: 304.1164, found: 304.1157.

***m*-tolyl diethylcarbamodithioate (4e)[3]**

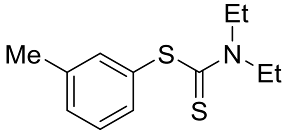

Purification by flash column chromatography on silica gel, R_f = 0.57; petroleum ether/ EtOAc = 10/1; isolated yield = 59% (14.2 mg, with TETD); yellow solid; M.p. 75-76 °C; ^{1}H NMR (400 MHz, $CDCl_3$) ppm: 7.41-7.33 (d, *J* = 8.0 Hz, 2H), 7.31-7.22 (d, *J* = 7.6 Hz, 2H), 4.10-3.96 (q, *J* = 6.9 Hz, 2H), 3.94-3.40 (q, *J* = 6.9 Hz, 3H), 2.45-2.36 (s, 3H), 1.45-1.36 (t, *J* = 7.0 Hz, 3H), 1.34-1.22 (t, *J* = 7.0 Hz, 3H); ^{13}C NMR (100 MHz, $CDCl_3$) ppm: 196.5, 140.3, 137.0, 130.0, 128.2, 50.0, 47.2, 21.5, 12.7, 11.6; IR (KBr) ν_{max} 2977, 2930, 1483, 1418, 1376, 1267, 1202, 1142, 1008, 978, 915, and 808 cm^{-1}.

3-methoxyphenyl diethylcarbamodithioate (4f)[5]

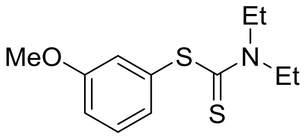

Purification by flash column chromatography on silica gel, R_f = 0.52; petroleum ether/ EtOAc = 10/1; isolated yield = 79% (20.3 mg, with TETD); deep yellow liquid; ^{1}H NMR (400 MHz, $CDCl_3$) ppm: 7.38-7.31 (t, *J* = 7.8 Hz, 1H), 7.12-7.06 (m, 1H), 7.06-7.02 (m, 1H), 7.02-6.97 (m, 1H), 4.10-3.97 (q, *J* = 6.9 Hz, 2H), 3.97-3.83 (m, 2H), 3.83-3.79 (m, 3H), 1.43-1.36 (t, *J* = 7.1 Hz, 3H), 1.32-1.27 (m, 3

H); ^{13}C NMR (100 MHz, $CDCl_3$) ppm: 195.7, 159.7, 132.4, 129.7, 129.3, 122.1, 116.2, 55.4, 49.8, 47.3, 12.8, 11.6; IR (KBr) ν_{max} 2973, 2931, 1591, 1577, 1461, 1415, 1269, 1231, 1206, 1143, 1010, 979, 917, 827, and 775 cm^{-1}.

2,5-dimethylphenyl diethylcarbamodithioate (4g)[6]

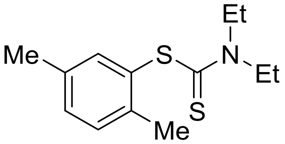

Purification by flash column chromatography on silica gel, R_f = 0.47; petroleum ether/EtOAc = 10/1; isolated yield = 58% (14.7 mg, with TETD); brown liquid; ^{1}H NMR (400 MHz, $CDCl_3$) ppm: 7.30-7.26 (m, 1H), 7.24-7.21 (m, 1H), 7.21-7.15 (m, 1H), 4.09-3.98 (m, 2H), 3.94-3.83 (q, J = 3.8 Hz, 2H), 2.41-2.34 (s, 1H), 2.34-2.30 (s, 1H), 1.48-1.36 (t, J = 7.0 Hz, 3H), 1.35-1.27 (m, 3H); ^{13}C NMR (100 MHz, $CDCl_3$) ppm: 195.1, 140.7, 138.2, 136.2, 131.5, 130.6, 130.5, 49.7, 47.3, 20.8, 20.4, 12.8, 11.7; IR (KBr) ν_{max} 3434, 2975, 2930, 1478, 1415, 1378, 1300, 1268, 1206, 1143, 1008, 979, 917, and 813.

4-chlorophenyl diethylcarbamodithioate (4h)[3]

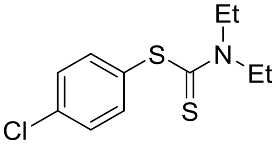

Purification by flash column chromatography on silica gel, R_f = 0.61; petroleum ether/ EtOAc = 15/1; isolated yield =85% (22.1 mg, with TETD); white solid; M.p. 90-91 °C; ^{1}H NMR (400 MHz, $CDCl_3$) ppm: 7.49-7.31 (s, 4H), 4.09-3.97 (q, J = 7.0 Hz, 2H), 3.93-3.79 (t, J = 7.0 Hz, 2H), 1.48-1.35 (t, J = 7.1 Hz, 3H), 1.35-1.26 (m, 3H); ^{13}C NMR (100 MHz, $CDCl_3$) ppm: 195.2, 138.4, 136.5, 130.0, 129.3, 50.0, 47.3, 12.8, 11.6; IR (KBr) ν_{max} 405, 2975, 1486, 1420, 1386, 1353, 1267, 1203, 1140, 1093, 915, and 823 cm^{-1}; HRMS (EI) calcd. for $C_{11}H_{14}ClNS_2$: $[M]^+$: 259.0256, found: 259.0252.

4-fluorophenyl diethylcarbamodithioate (4i)[3]

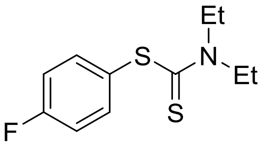

Purification by flash column chromatography on silica gel, R_f = 0.55; petroleum ether/EtOAc = 10/1; isolated yield = 60% (14.7 mg, with TETD); yellow liquid; ^{1}H NMR (400 MHz, $CDCl_3$) ppm: 7.50-7.40 (m, 2H),7.17-7.08 (m, 2H), 4.10-3.96 (q, J = 7.0 Hz, 2H), 3.91-3.39 (q, J = 7.0 Hz, 2H), 1.45-1.37 (t, J = 7.0 Hz, 3H), 1.32-1.26 (m, 3H); ^{13}C NMR (100 MHz, $CDCl_3$) ppm: 195.8, 165.2, 162.7, 139.3 (139.3, 139.2, d, J = 8.7 Hz), 127.0 (127.0,127.0, d, J = 3.4 Hz), 116.3 (116.4, 116.2, d, J = 21.9 Hz),

50.0, 47.3, 12.7, 11.6; IR (KBr) ν_{max} 2983, 2929, 1588, 1486, 1420, 1394, 1295, 1204, 1140, 1073, 97 8, 915, and 832 cm^{-1}.

3-chlorophenyl diethylcarbamodithioate (4j)[3]

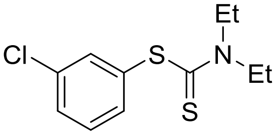

Purification by flash column chromatography on silica gel, R_f = 0.61; petroleum ether/ EtOAc = 15/1; isolated yield = 60% (15.7 mg, with TETD); brown liquid; ^{1}H NMR (400 MHz, $CDCl_3$) ppm: 7.55-7.47 (m, 1H), 7.47-7.40 (m, 1H), 7.40-7.32 (m, 2H), 4.10-3.97 (q, J = 6.9 Hz, 2H), 3.88-3.79 (q, J = 7.0 Hz, 2H), 1.44-1.36 (t, J = 7.0 Hz, 3H), 1.32-1.27 (m, 3H); ^{13}C NMR (100 MHz, $CDCl_3$) ppm: 194.7, 136.9, 135.4, 134.3, 133.2, 130.1, 130.0, 50.0, 47.4, 12.8, 12.6; IR (KBr) ν_{max} 3432, 2923, 1562, 1504, 1459, 1400, 1380, 1249, 1151, 1067, 980, 864, and 790 cm^{-1}.

3-fluorophenyl diethylcarbamodithioate (4k)[5]

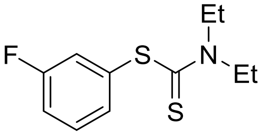

Purification by flash column chromatography on silica gel (R_f = 0.46, petroleum ether/EtOAc = 10/1); isolated yield = 66% (16.1 mg, with TETD); yellow liquid; ^{1}H NMR (400 MHz, $CDCl_3$) ppm: 7.44-7.36 (m, 1H), 7.30-7.26 (m, 1H), 7.25-7.20 (m, 1H), 7.20-7.12 (m, 1H), 4.09-3.97 (q, J = 7.0 Hz, 2H), 3.39-3.80 (m, 2H), 1.48-1.37 (t, J = 7.0 Hz, 3H), 1.32-1.27 (t, J = 7.0 Hz, 3H); ^{13}C NMR (100 MHz, $CDCl_3$) ppm: 194.8, 163.7, 161.2, 133.1 (133.3, 133.2, 132.9, 132.9, m), 130.1 (130.2, 130.1, d, J = 8.1 Hz), 124.1 (124.2, 124.0, d, J = 22.1 Hz), 117.2 (117.3, 117.1, d, J = 21.5 Hz), 49.9, 47.4, 12.8, 11.6; IR (KBr) ν_{max} :2971, 2931, 1257, 1488, 1475, 1415, 1271, 1206, 1050, 880, and 783 cm^{-1}.

4-vinylphenyl diethylcarbamodithioate (4l)

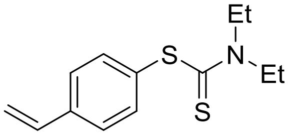

Purification by flash column chromatography on silica gel, R_f = 0.45; petroleum ether/EtOAc = 15/1; isolated yield = 73% (18.4 mg, with TETD); yellow liquid; ^{1}H NMR (400 MHz, $CDCl_3$) ppm: 7.51-7. 45 (m, 2H), 7.45-7.39 (m, 2H), 6.84-6.68 (m, 1H), 5.93-5.77 (m, 1H), 5.43-5.27 (d, J = 10.9 Hz, 1H), 4.10-3.94 (q, J = 7.0 Hz, 2H), 3.94-3.78 (q, J = 7.0 Hz, 2H), 1.47-1.35 (q, J = 7.1 Hz, 3H), 1.31-1.2 6 (m, 3H); ^{13}C NMR (100 MHz, $CDCl_3$) ppm: 195.9, 139.1, 137.2, 136.2, 130.6, 126.9, 115.5, 49.9,

47.3, 12.8, 11.6; IR (KBr) ν_{max} 3431, 2970, 2923, 1488, 1412, 1355, 1271, 1201, 1136, 1065, 974, 91 4, and 828 cm^{-1}; HRMS (EI) calcd. for $C_{13}H_{17}NS_2$: $[M]^+$: 251.0802, found: 251.0802.

naphthalen-1-yl diethylcarbamodithioate (4m)

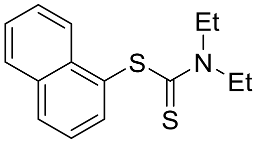

Purification by flash column chromatography on silica gel, R_f = 0.53; petroleum ether/EtOAc = 15/1; isolated yield = 59% (16.3 mg, with TETD); white solid; M.p. 74-75 °C; ^{1}H NMR (400 MHz, $CDCl_3$) ppm: 8.27-8.19 (d, J = 8.2 Hz, 1H), 8.04-7.95 (d, J = 8.2 Hz, 1H), 7.93-7.85 (m, 1H), 7.80-7.72 (m, 1H), 7.59-7.45 (m, 3H), 4.11-3.92 (m, 4H), 1.56-1.47 (m, 3H), 1.37-1.24 (m, 3H); ^{13}C NMR (100 M Hz, $CDCl_3$) ppm: 194.9, 137.2, 135.3, 134.2, 131.4, 128.9, 128.7, 127.2, 126.3, 125.9, 125.8, 49.8, 47. 4, 13.0, 11.7; IR (KBr) ν_{max} 3436, 2928, 1484, 1419, 1266, 1204, 1139, 1066, 1005, and 736 cm^{-1}; H RMS (ESI) calcd. for $C_{15}H_{17}NS_2$: $[M+Na]^+$: 298.0695, found: 298.0693.

furan-2-yl diethylcarbamodithioate (4n)

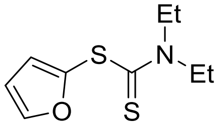

Purification by flash column chromatography on silica gel, R_f = 0.46; petroleum ether/EtOAc = 10/1; isolated yield = 47% (10.2 mg, with TETD); yellow liquid; ^{1}H NMR (400 MHz, $CDCl_3$) ppm: 7.67-7.62(dd, J =5.3 Hz, 1H), 7.27-7.23 (m, 1H), 7.17-7.12 (m, 1H), 4.06-3.96 (q, J =7.0 Hz, 2H), 3.90-3. 81 (q, J = 7.0 Hz, 2H), 1.44-1.36 (t, J = 7.1 Hz,3H),1.32-1.27 (m, 3H); ^{13}C NMR (100 MHz, $CDCl_3$) ppm: 195.8, 138.9, 133.4, 129.2, 127.9, 50.4, 47.2, 12.9, 11.5; IR (KBr) ν_{max} 3434, 2974, 2929, 1488, 1417, 1383, 1269, 1203, 1144, 1066, 1012, 974, 915, and 706 cm^{-1}.

Phenyl Dibutylcarbamodithioate (4o)

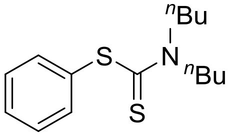

Purification by flash column chromatography on silica gel, R_f = 0.40; petroleum ether/EtOAc = 20/1; isolated yield = 46% (13.0 mg, with TBTD); brown liquid; ^{1}H NMR (400 MHz, $CDCl_3$) ppm: 7.56-7.38 (m, 5H), 4.03-3.38 (t, J = 7.8 Hz, 2H), 3.38-3.20 (t, J = 7.8 Hz, 2H),1.89-1.77 (m, 2H), 1.77-1.68 (m, 2H), 1.50-1.39 (m, 2H), 1.39-1.30 (m, 2H), 1.07-0.98 (t, J = 7.3 Hz, 3H), 0.98-0.89 (t, J = 7.3 Hz, 3H); ^{13}C NMR (100 MHz, $CDCl_3$) ppm: 196.3, 137.1, 131.8, 130.0, 129.0, 55.4, 53.1, 29.6,

28.4, 20.2, 13.9, 13.8; IR (KBr) ν_{max} 3420, 2959, 2930, 2872, 1484, 1440, 1413, 1367, 1290, 1249, 1219, 1183, 1091, and 744 cm^{-1}; HRMS (EI) calcd. for $C_{15}H_{23}NS_2$: $[M]^+$: 81.1272; found: 281.1265.

p-tolyl dibutylcarbamodithioate (4p)

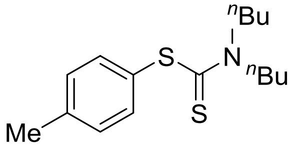

Purification by flash column chromatography on silica gel, R_f = 0.50; petroleum ether/EtOAc = 20/1; isolated yield = 52% (15.4 mg, with TBTD); yellow solid; ^{1}H NMR (400 MHz, $CDCl_3$) ppm: 7.39-7. 33 (d, J = 8.0 Hz, 2H), 7.26-7.21 (m, 2H), 3.99-3.88 (t, J = 7.8 Hz, 2H), 3.84-3.71 (t, J = 7.8 Hz, 2H), 2.49-2.35 (s, 3H), 1.89-1.77 (m, 2H), 1.77-1.67 (m, 2H), 1.51-1.39 (m, 2H), 1.39-1.21 (m, 2H), 1.09-0.98 (t, J = 7.3 Hz, 3H), 0.68-0.90 (t, J = 7.3 Hz, 3H); ^{13}C NMR (100 MHz, $CDCl_3$) ppm: 196.8, 140. 2, 137.0, 129.9,128.3, 55.3, 45.8, 41.9; IR (KBr) ν_{max} 23420, 2958, 2929, 2871, 1483, 1455, 1412, 136 7, 1144, 1219, 1249, 1144, 1092, 983, and 805 cm^{-1}; HRMS (EI) calcd. for $C_{16}H_{25}NS_2$: $[M]^+$: 295.14 28; found: 295.1421.

4-methoxyphenyl dibutylcarbamodithioate (4q)

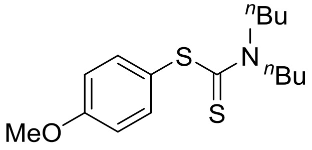

Purification by flash column chromatography on silica gel, R_f = 0.56; petroleum ether/EtOAc = 20/1; isolated yield = 59% (18.3 mg, with TBTD); yellow soild; M.p. 45-46 °C; ^{1}H NMR (400 MHz, CDC l_3) ppm: 7.42-7.33 (m, 2H), 6.99-6.92 (m, 2H), 4.00-3.89 (t, J = 7.9 Hz, 2H), 3.88-3.82 (s, 3H), 3.79-3.71 (t, J = 7.9 Hz, 2H), 1.88-1.78 (m, 2H), 1.78-1.67 (m, 2H), 1.49-1.38 (m, 2H), 1.38-1.31 (m, 2H), 1.08-0.98 (t, J = 7.3 Hz, 3H), 0.99-0.91 (t, J = 7.3 Hz, 3H); ^{13}C NMR (100 MHz, $CDCl_3$) ppm: 197.4, 161.0, 138.6, 122.6, 114.6, 55.5, 55.3, 52.9, 29.6, 28.4, 20.2, 13.9, 13.8; IR (KBr) ν_{max} 958, 2931, 281 7, 1591, 1493, 1459, 1412, 1292, 1250, 1219, 1172, 1031, and 824, cm^{-1}; HRMS (EI) calcd. for $C_{16}H_{25}NOS_2$: $[M]^+$: 311.1378, found: 311.1370.

2,2-diphenylvinyl dimethylcarbamodithioate (6)

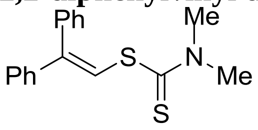

Purification by flash column chromatography on silica gel, R_f = 0.33; petroleum ether/EtOAc = 10/1; isolated yield = 29% (17.3 mg); yellow viscous liquid; ^{1}H NMR (400 MHz, $CDCl_3$) ppm: 7.79-7.67 (s, 1H), 7.46-7.31 (m, 6H), 7.30-7.23 (m, 4H), 3.62-3.48 (s, 3H), 3.38-3.21 (s, 3H),; ^{13}C NMR (100

MHz, $CDCl_3$) ppm: 194.4, 142.0, 141.1, 139.8, 129.7, 128.4, 128.3, 128.0, 127.8, 127.6, 123.5, 45.4, 41.6, 29.7, 22.7; HRMS (EI) calcd. for $C_{17}H_{17}NS_2$: $[M]^+$: 299.0802, found: 299.0801.

5 References

1. Yu, J., Liu, J., Shi, G., Shao, C., Zhang, Y. (2015). Ligand-Promoted Oxidative Cross-Coupling of Aryl Boronic Acids and Aryl Silanes by Palladium Catalysis. *Angew. Chem. Int. Ed.* 54, 4079-4082. doi: 10.1002/ange.201412288.
2. Lu, M., Ding, X., Shao, C., Hu, Z., Luo, H., Zhi, S., Hu, H., Kan, Y., Loh, T. -P. (2020). Direct Hiyama Cross-Coupling of (Hetero)arylsilanes with C(sp^2)-H Bonds Enabled by Cobalt Catalysis. *Org. Lett.* 22, 2663-2668. doi: 10.1021/acs.orglett.0c00631.
3. Wu, X. M., Yan, G. B. (2019). Copper-Catalyzed Synthesis of S-Aryl Dithiocarbamates from Tetraalkylthiuram Disulfides and Aryl Iodides in Water. *Synlett*. 30, 610-614. doi: 10.1055/s-0037-1612086.
4. Xu, W., Gao, F., Dong, Z. B. (2018). Copper-Catalyzed S-Arylation Starting from Arylboronic Acids and Tetraalkylthiuram Disulfide. *Eur. J. Org. Chem.* 2018, 821-828. doi: 10.1002/ejoc.201701757.
5. Qi, C., Guo, T., Xiong, W. (2016). Copper-Mediated Coupling of Boronic Acids, Amines, and Carbon Disulfide: An Approach to Organic Dithiocarbamates. *Synlett*. 27, 2626-2630. doi: 10.1055/s-0035-1560561.
6. Zeng, M. T., Xu, W., Liu, X., Chang, C. -Z., Zhu, H., Dong, Z. -B. (2017). Copper-Catalyzed S-Arylation of Tetraalkylthiuram Disulfides by Using Diaryliodonium Salts. *Eur. J. Org. Chem.* 2017, 6060-6066. doi:10.1002/ejoc.201701056.

6 Copy of ^{1}H and ^{13}C NMR Spectra

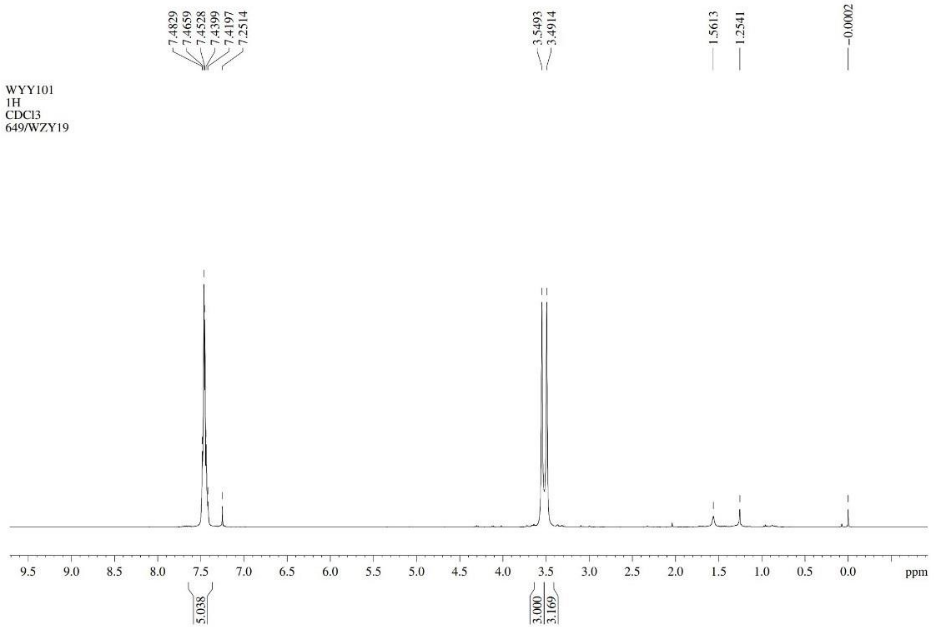

^{1}H NMR spectrum of compound **3a**

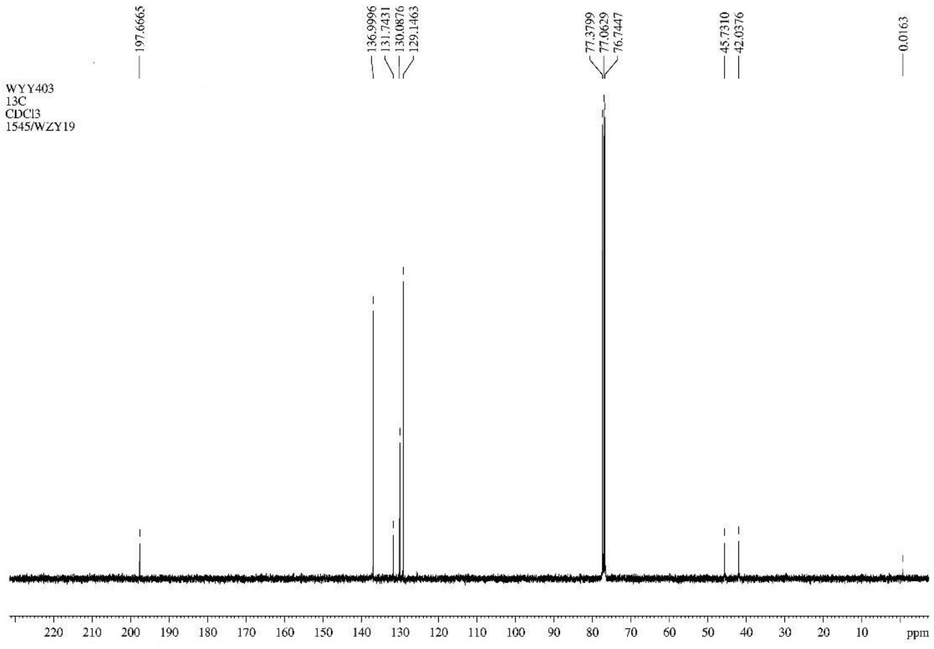

^{13}C NMR spectrum of compound **3a**

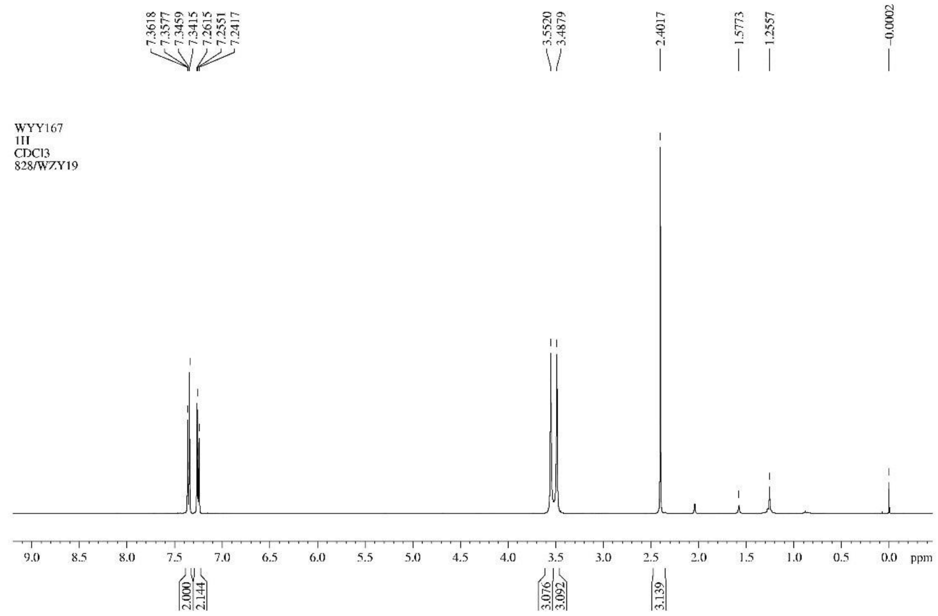

^{1}H NMR spectrum of compound **3b**

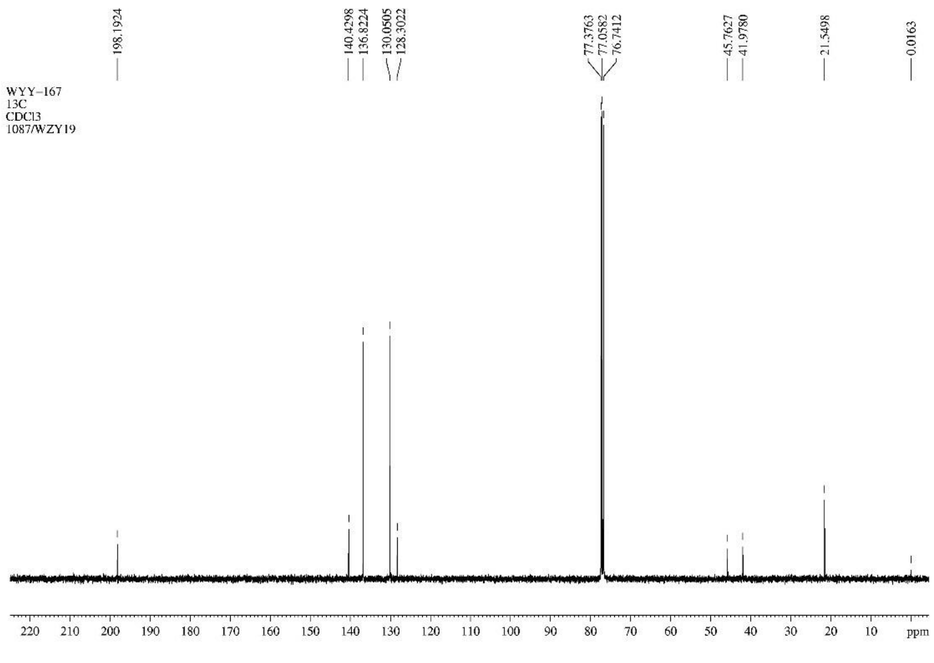

^{13}C NMR spectrum of compound **3b**

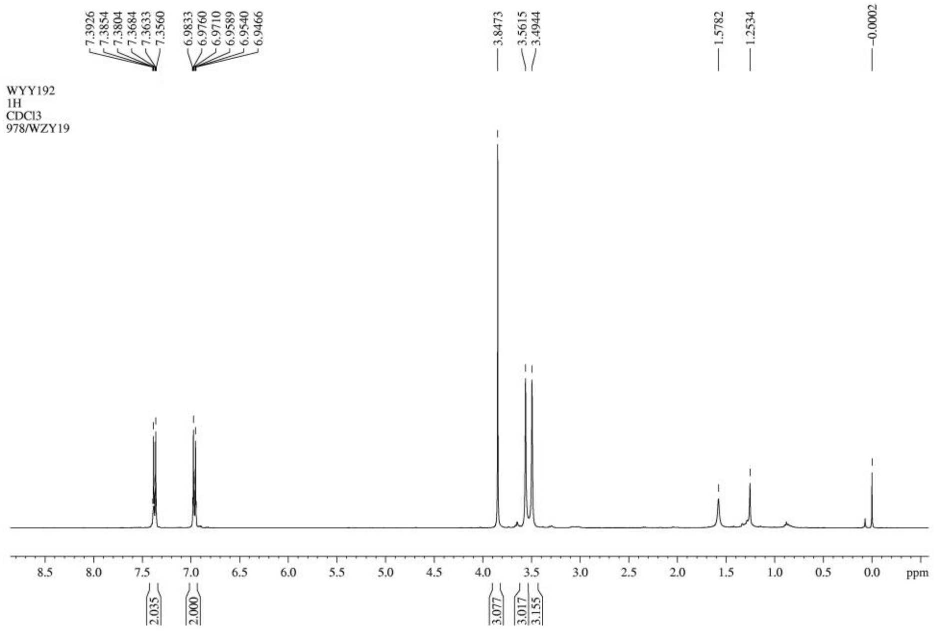

^{1}H NMR spectrum of compound **3c**

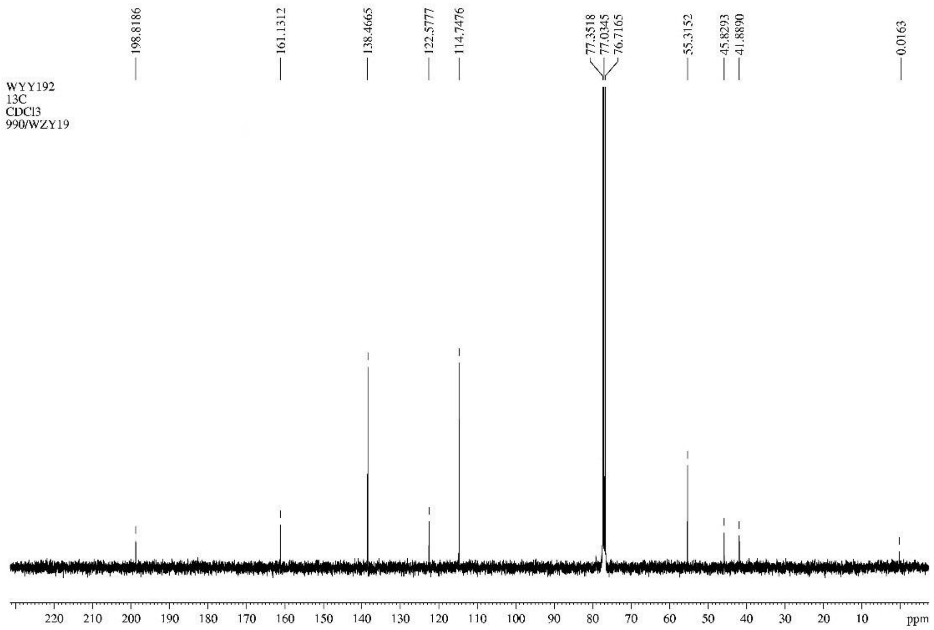

^{13}C NMR spectrum of compound **3c**

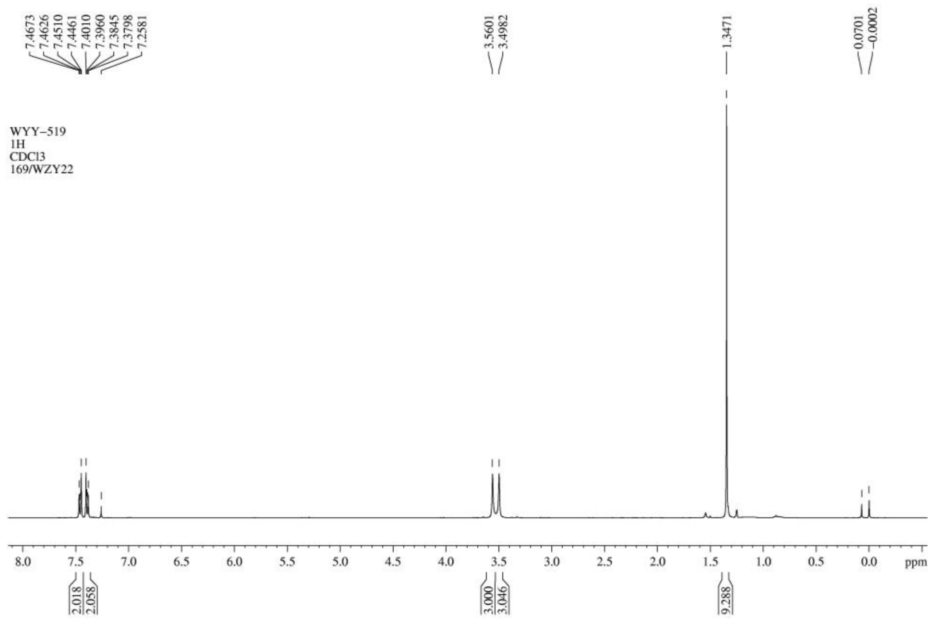

^{1}H NMR spectrum of compound **3d**

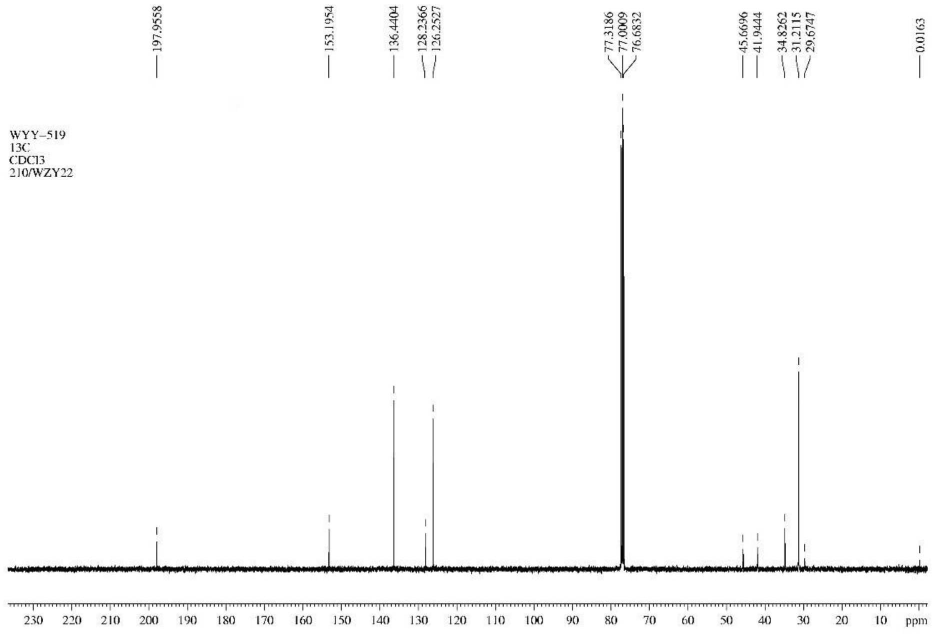

^{13}C NMR spectrum of compound **3d**

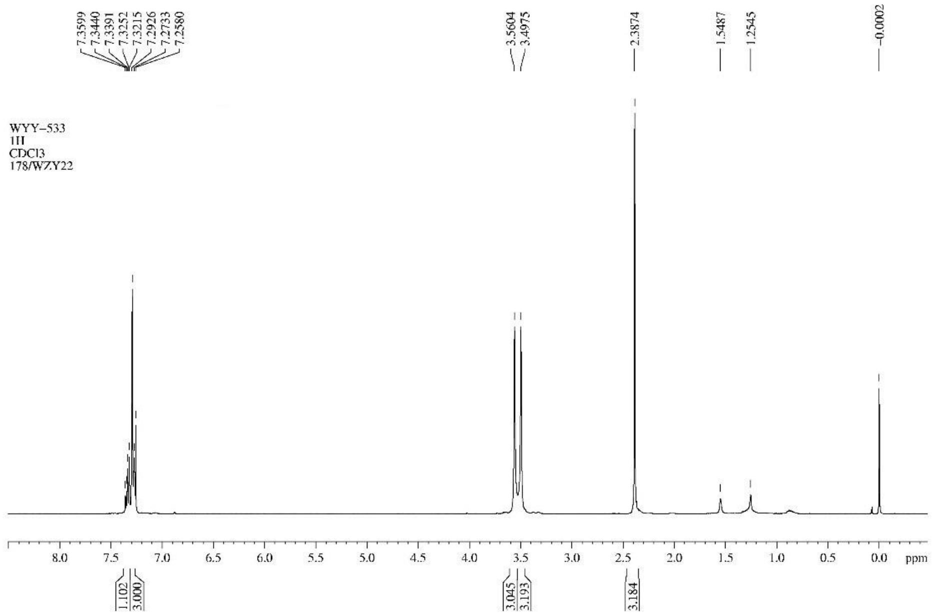

^{1}H NMR spectrum of compound **3e**

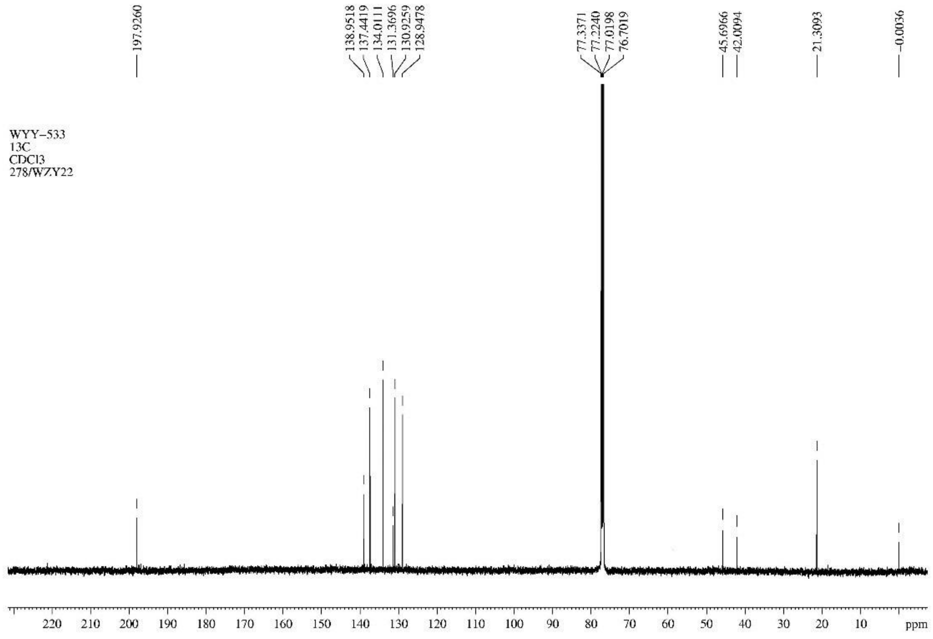

^{13}C NMR spectrum of compound **3e**

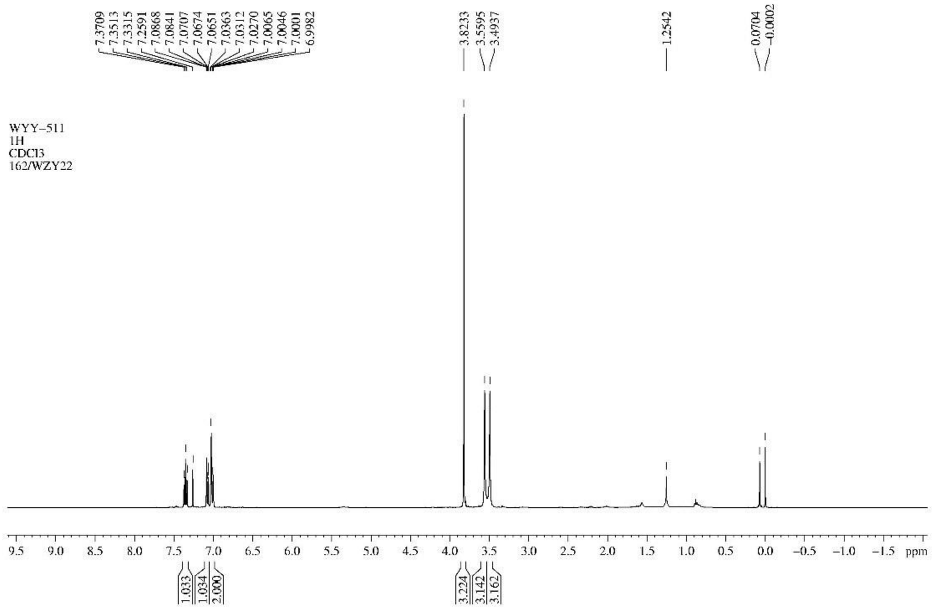

^{1}H NMR spectrum of compound **3f**

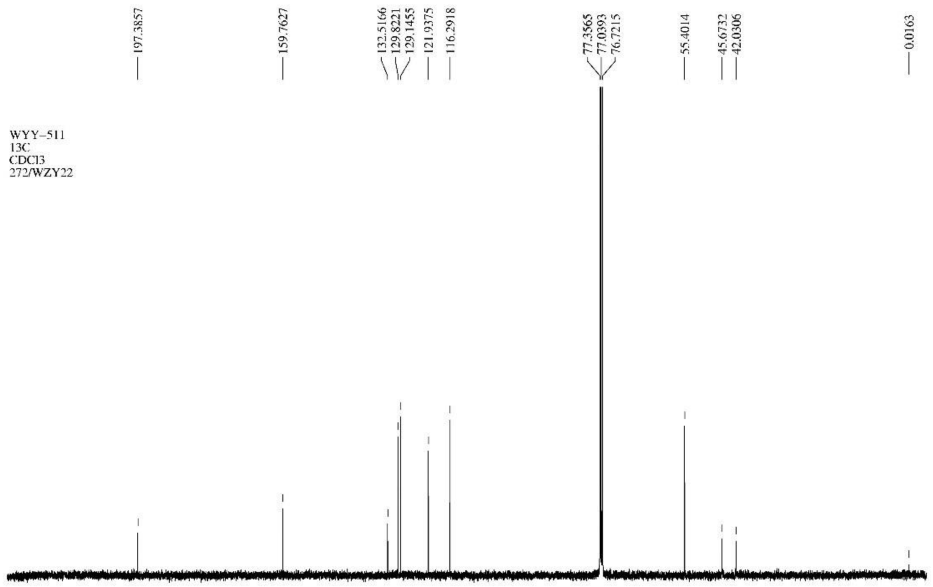

^{13}C NMR spectrum of compound **3f**

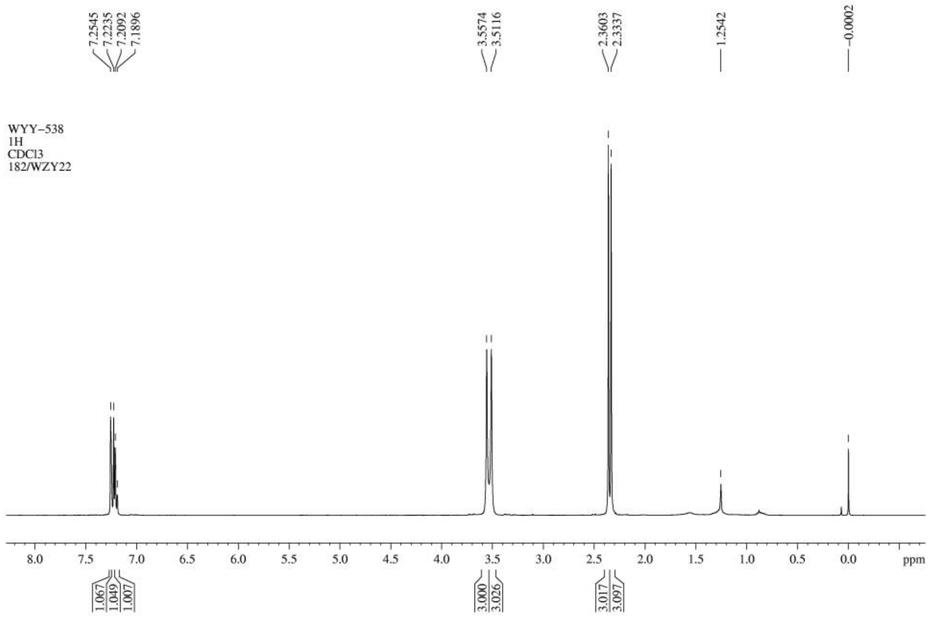

^{1}H NMR spectrum of compound **3g**

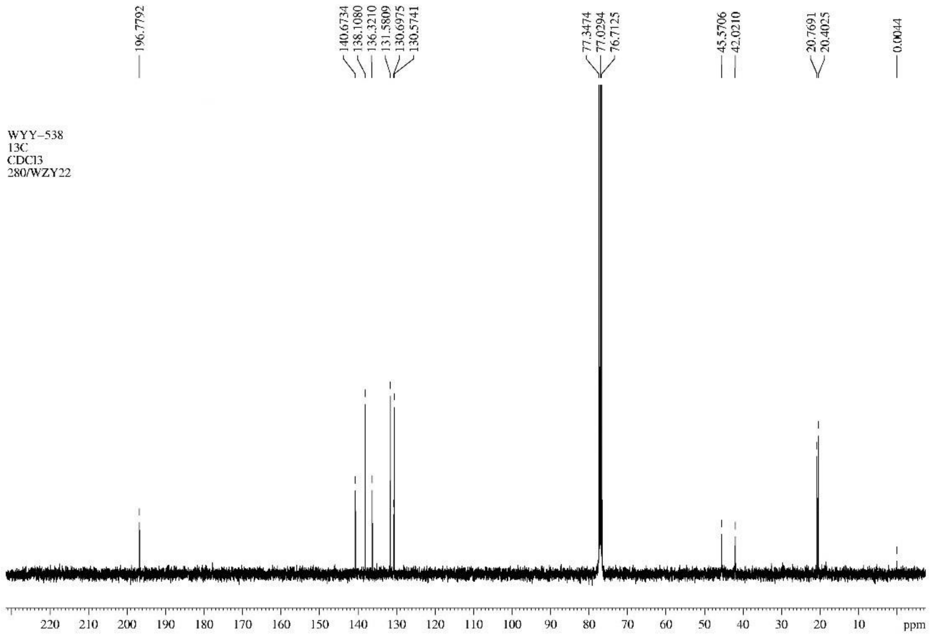

^{13}C NMR spectrum of compound **3g**

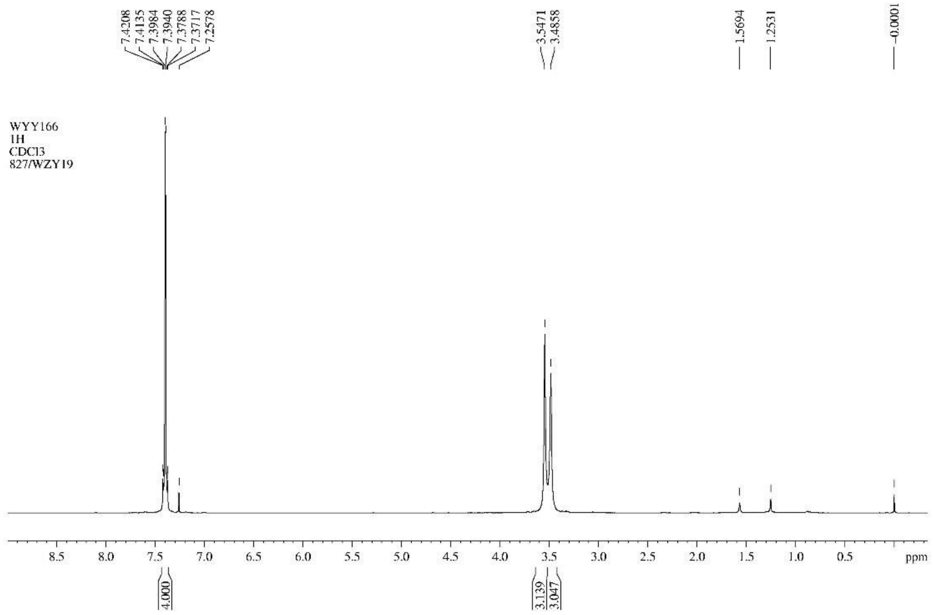

^{1}H NMR spectrum of compound **3h**

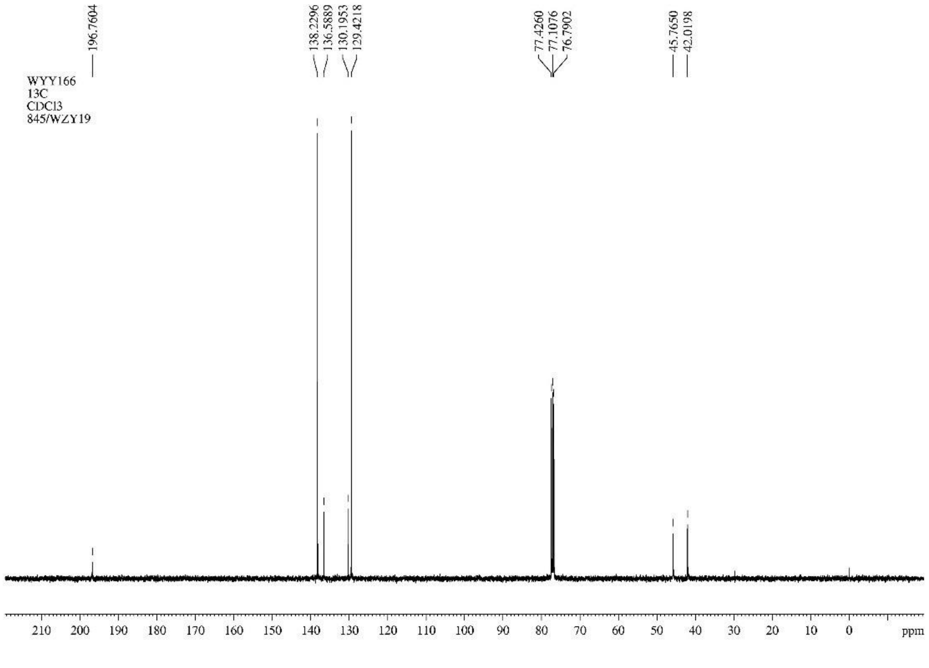

^{13}C NMR spectrum of compound **3h**

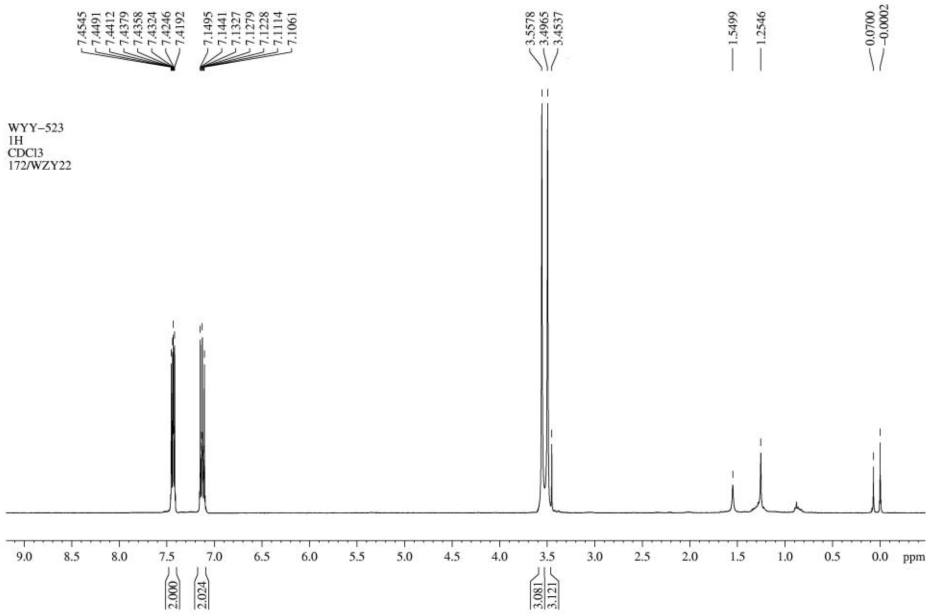

^{1}H NMR spectrum of compound **3i**

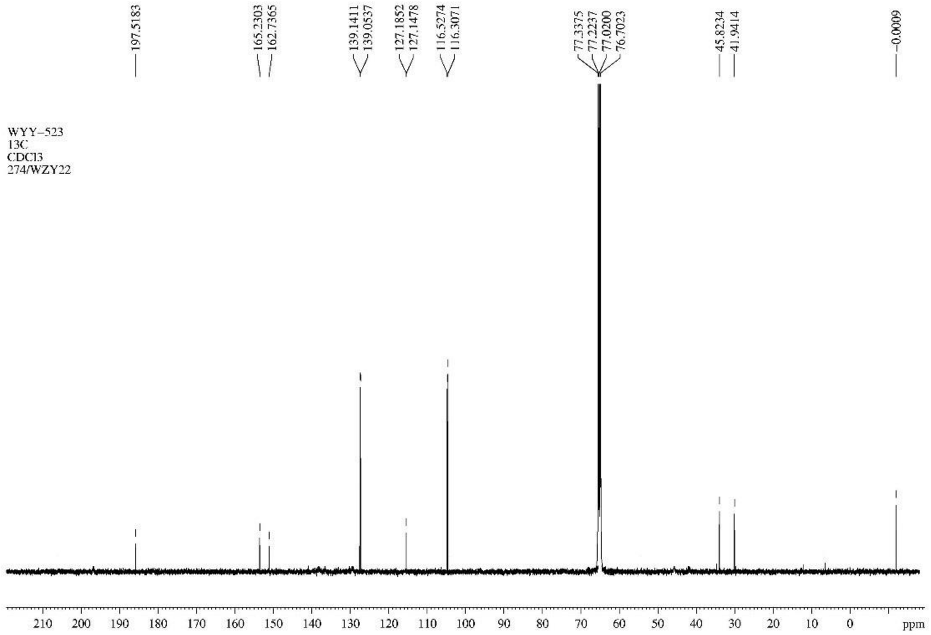

^{13}C NMR spectrum of compound **3i**

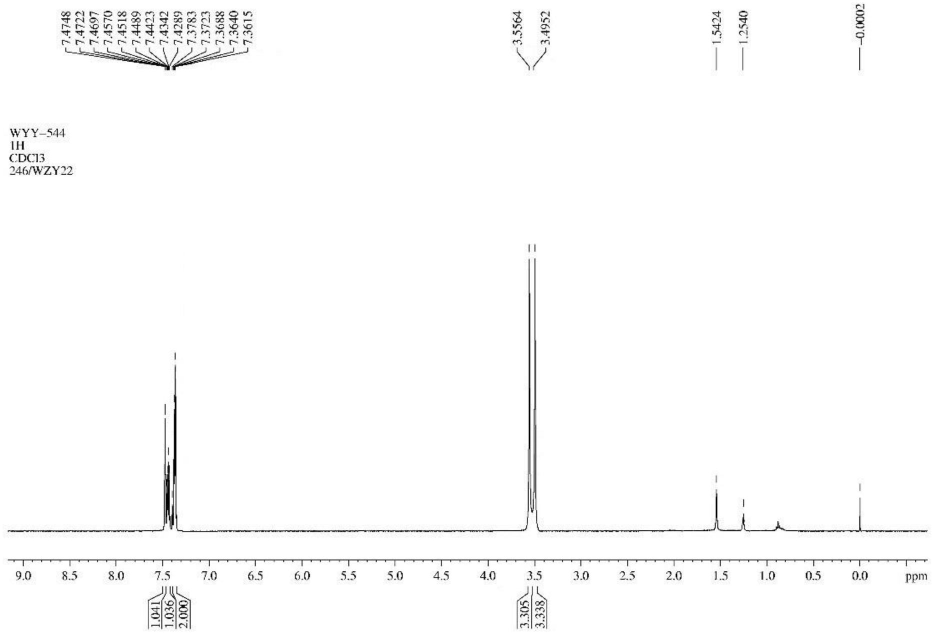

^{1}H NMR spectrum of compound **3j**

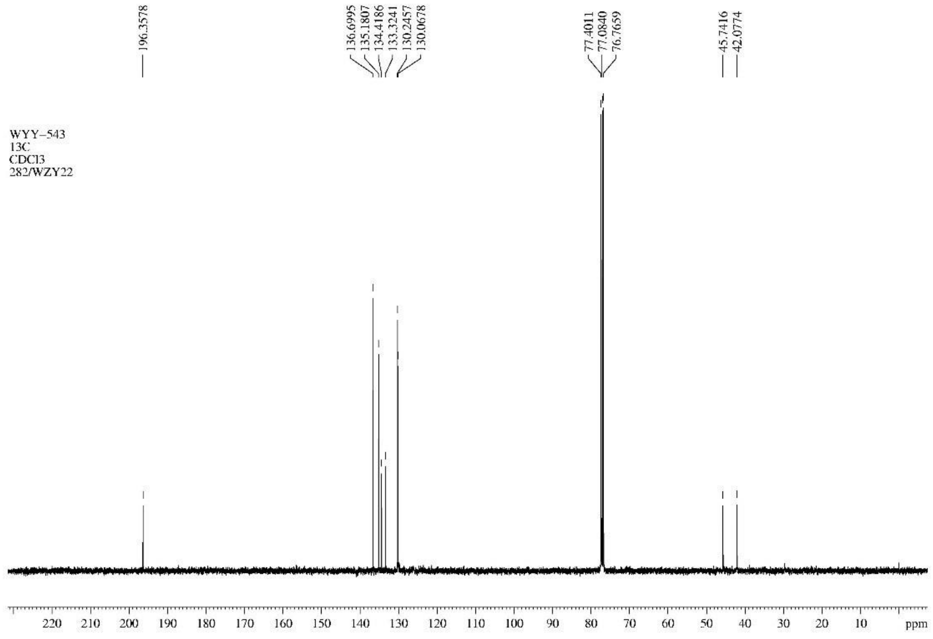

^{13}C NMR spectrum of compound **3j**

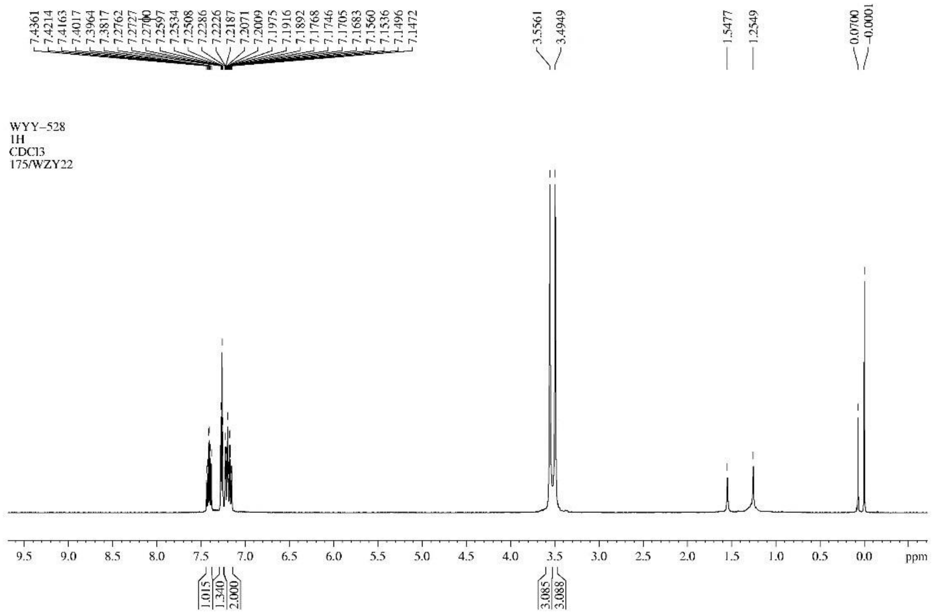

^{1}H NMR spectrum of compound **3k**

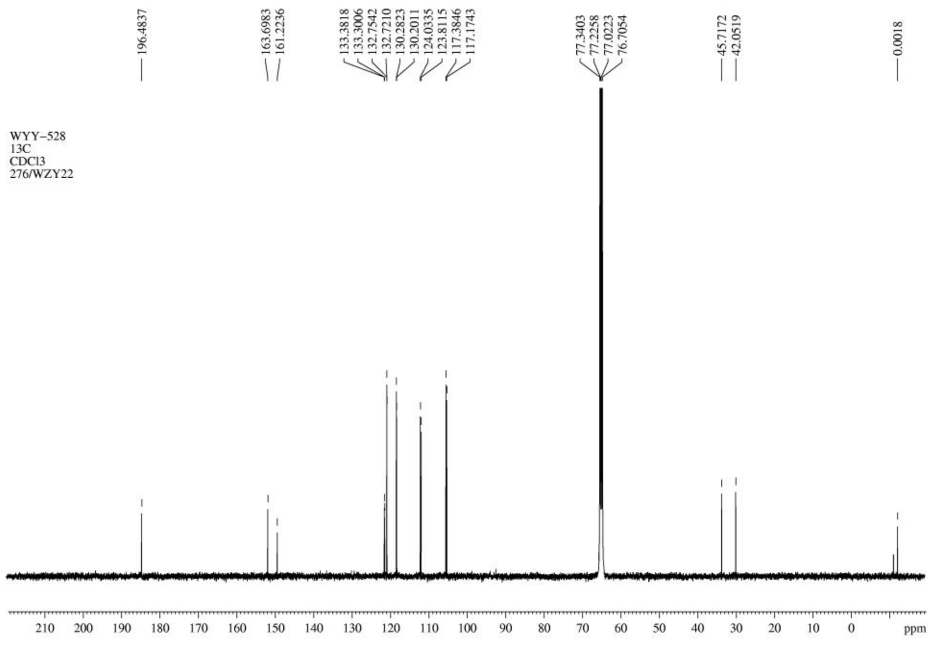

^{13}C NMR spectrum of compound **3k**

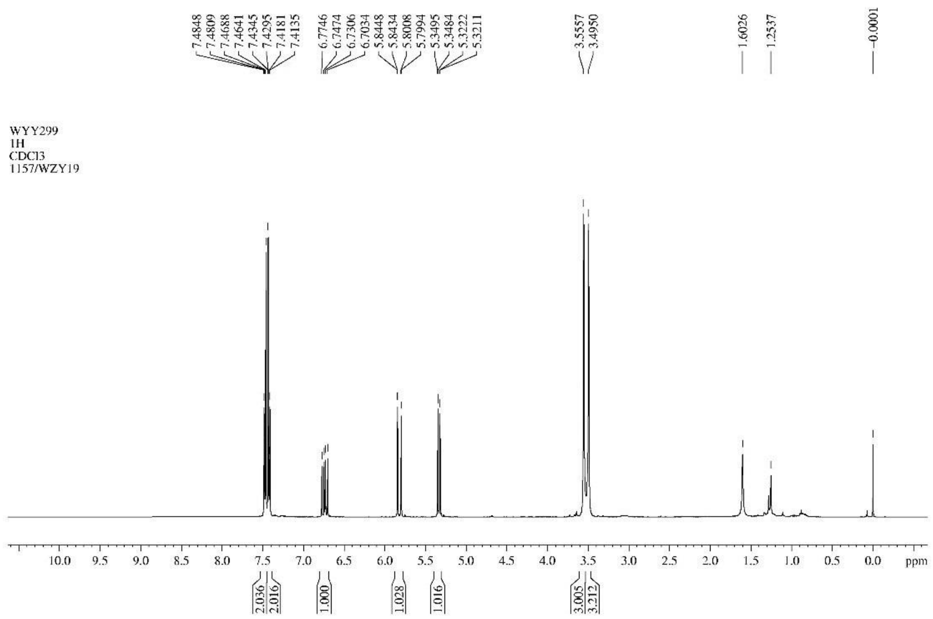

^{1}H NMR spectrum of compound **3l**

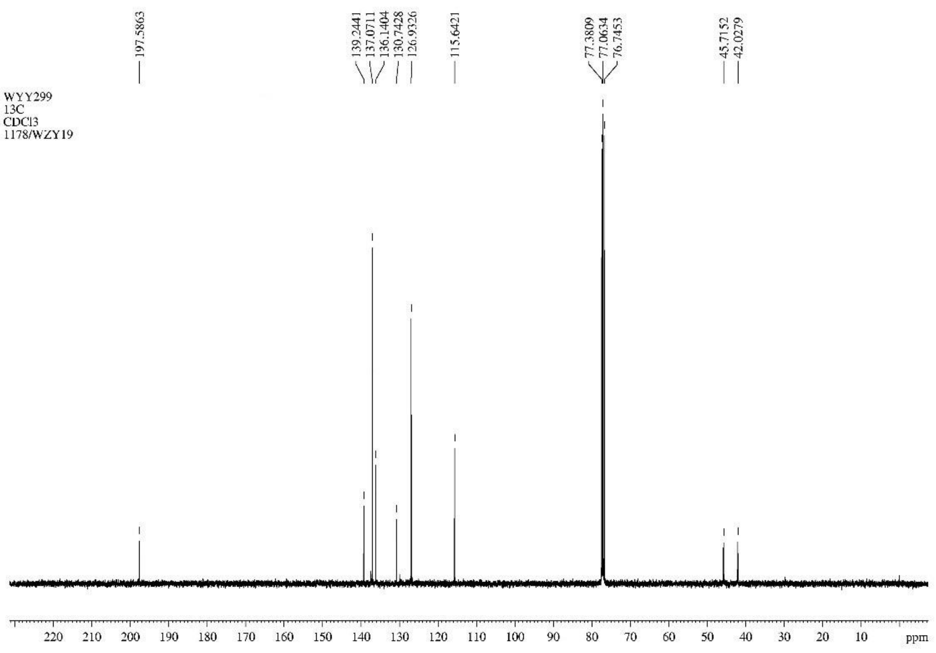

^{13}C NMR spectrum of compound **3l**

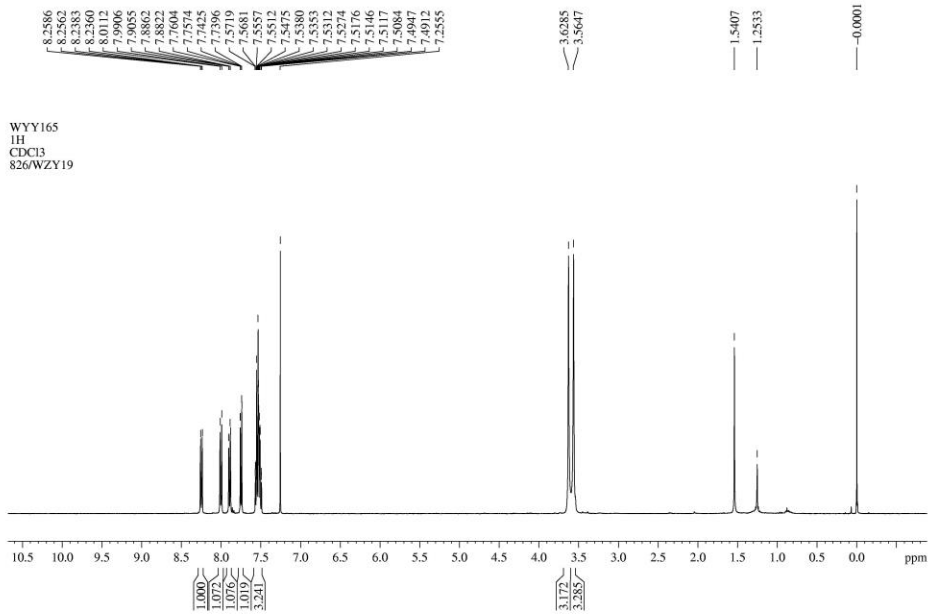

^{1}H NMR spectrum of compound **3m**

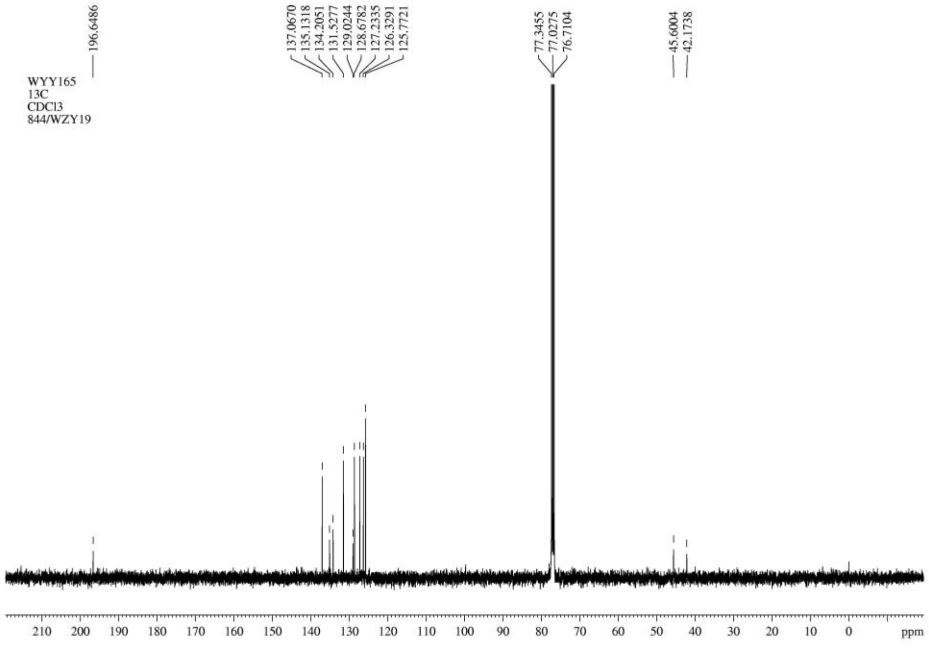

^{13}C NMR spectrum of compound **3m**

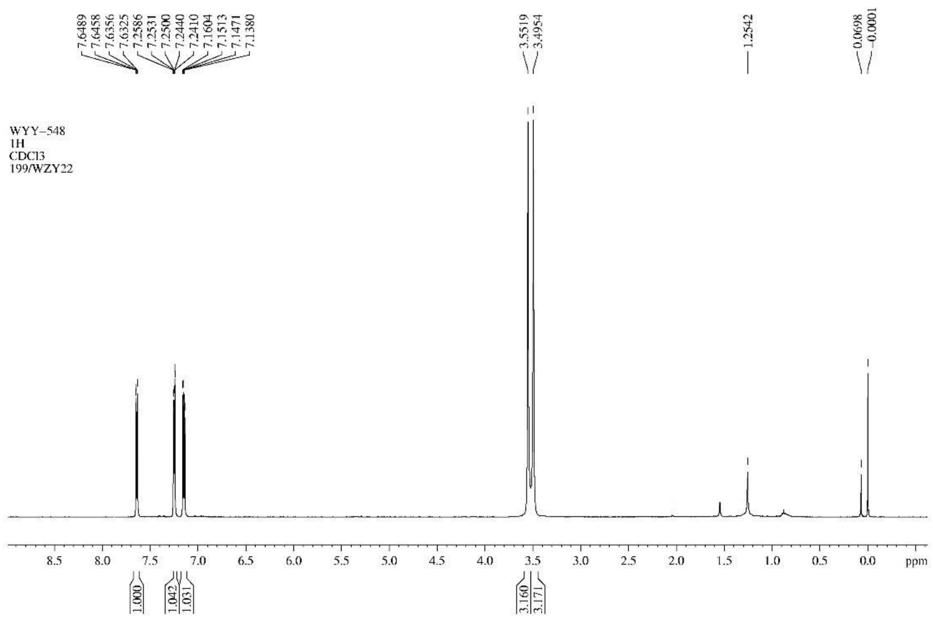

^{1}H NMR spectrum of compound **3n**

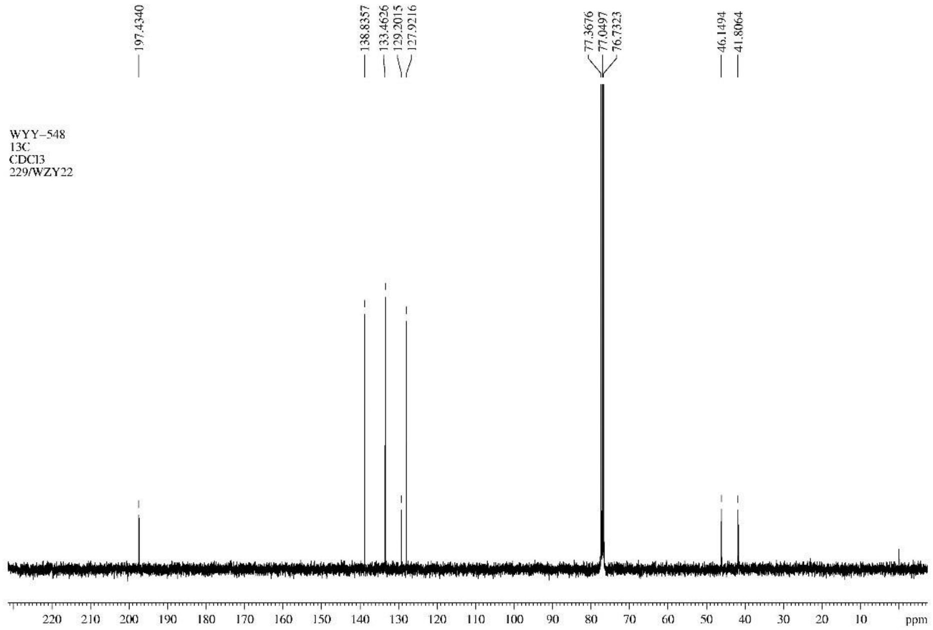

^{13}C NMR spectrum of compound **3n**

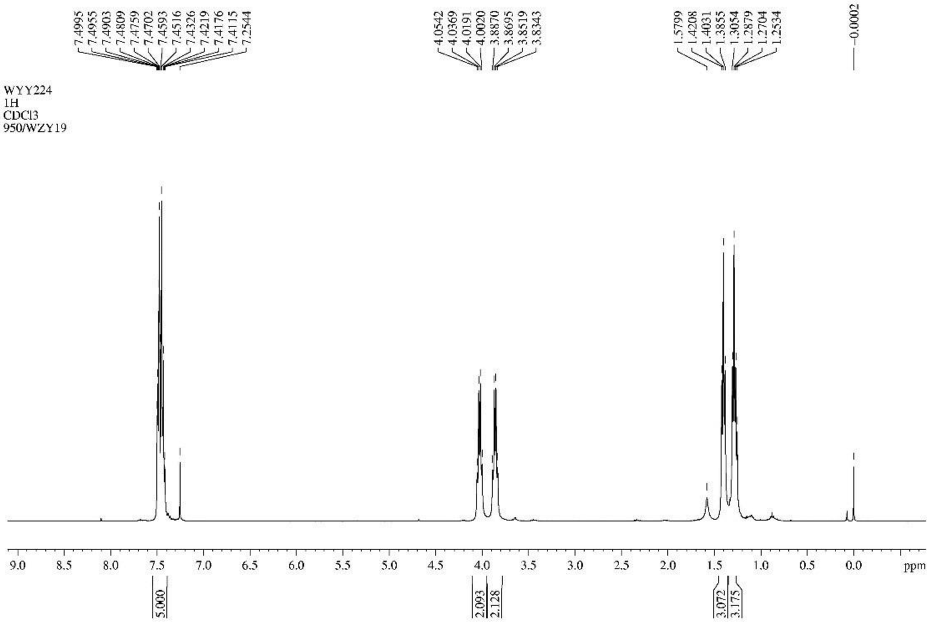

^{1}H NMR spectrum of compound **4a**

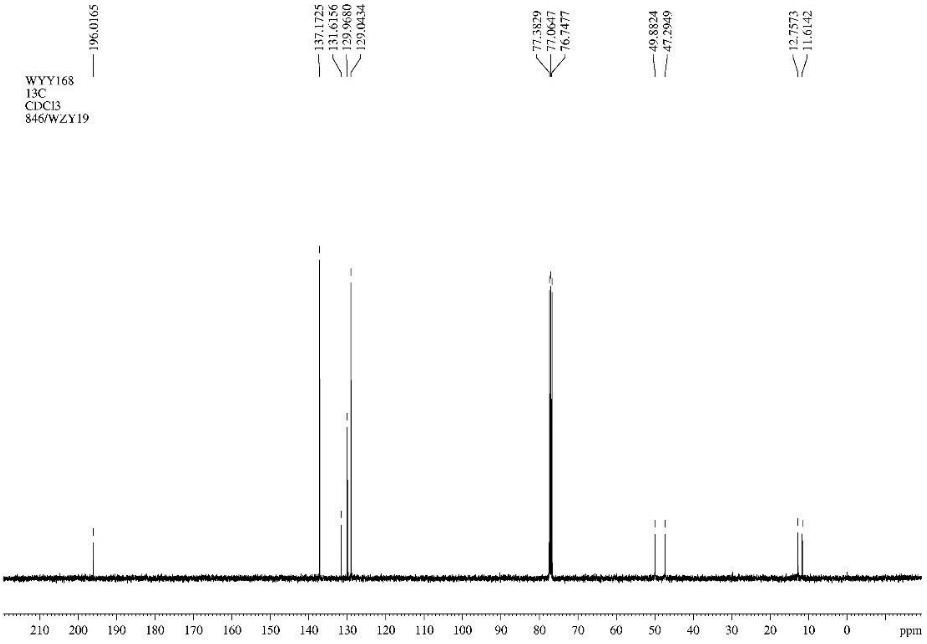

^{13}C NMR spectrum of compound **4a**

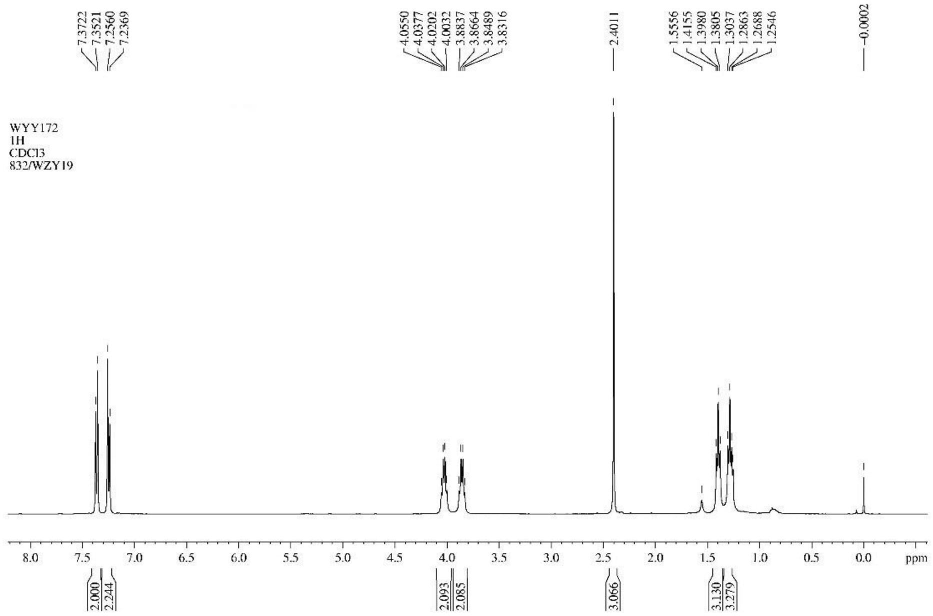

^{1}H NMR spectrum of compound **4b**

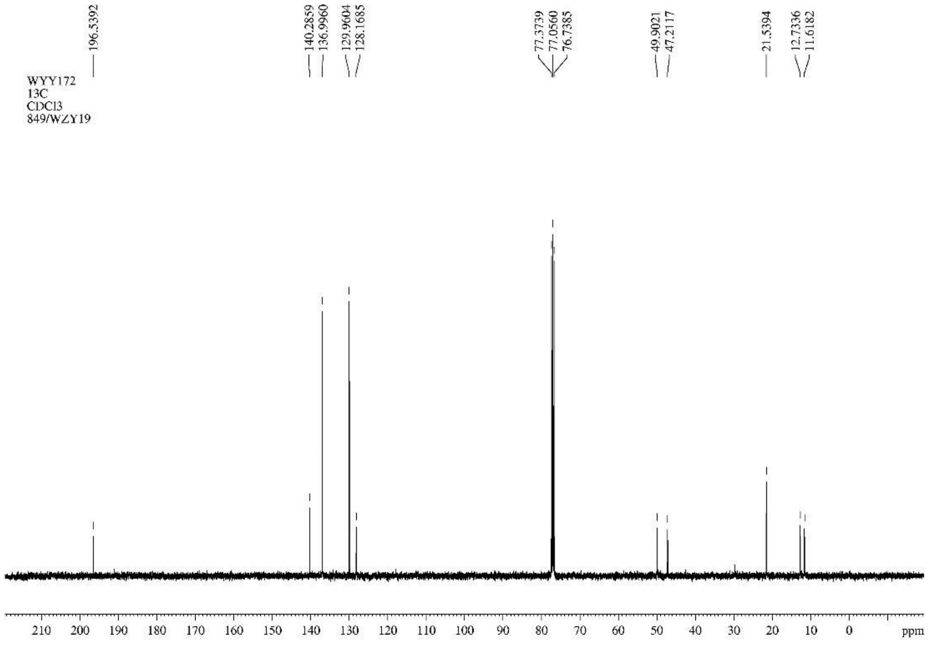

^{13}C NMR spectrum of compound **4b**

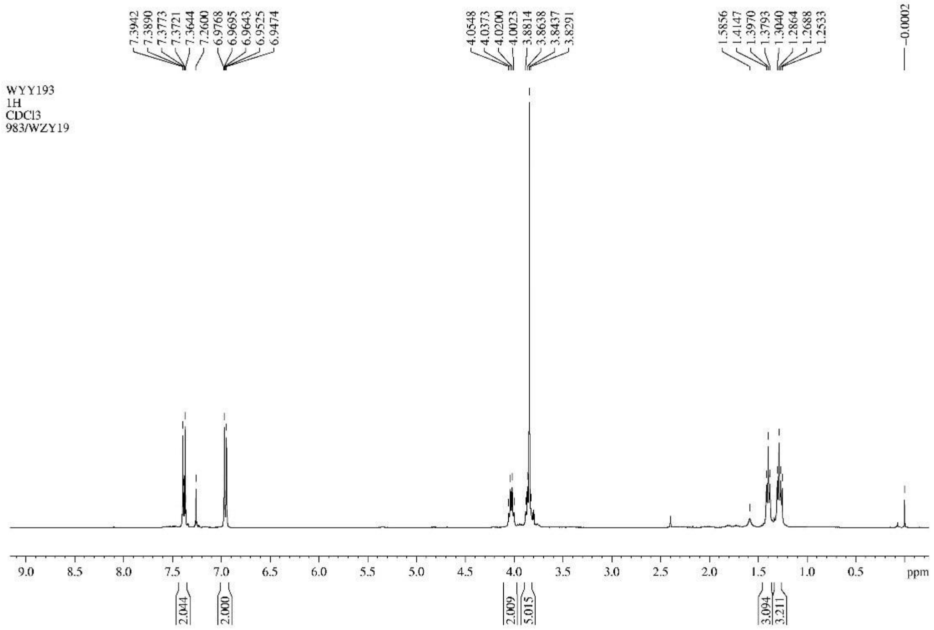

^{1}H NMR spectrum of compound **4c**

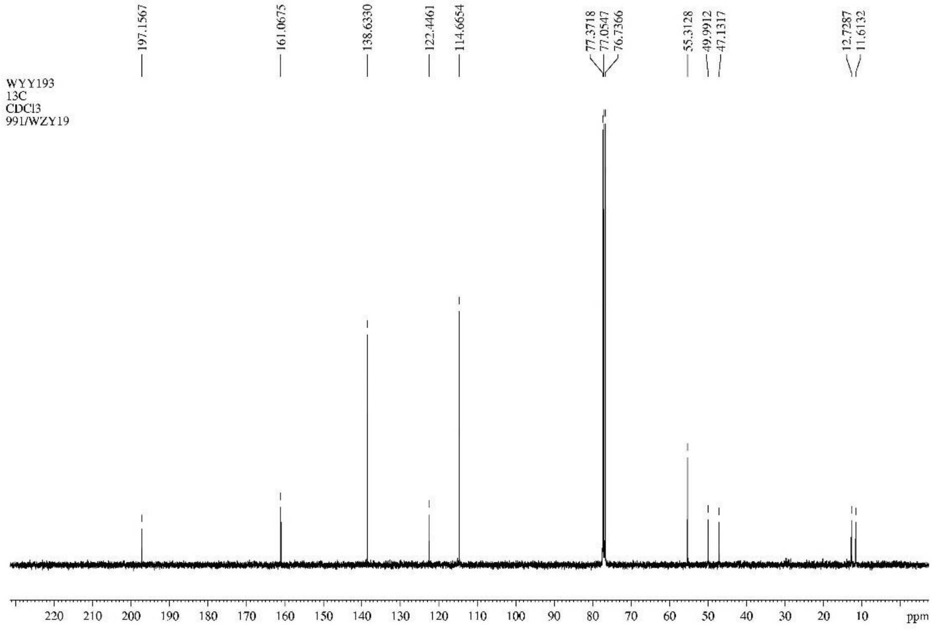

^{13}C NMR spectrum of compound **4c**

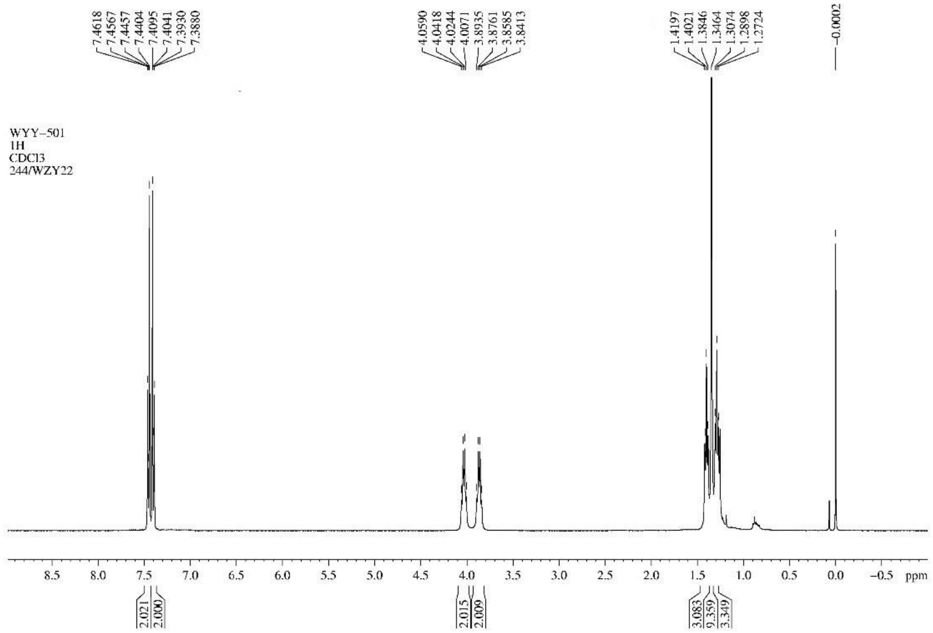

^{1}H NMR spectrum of compound **4d**

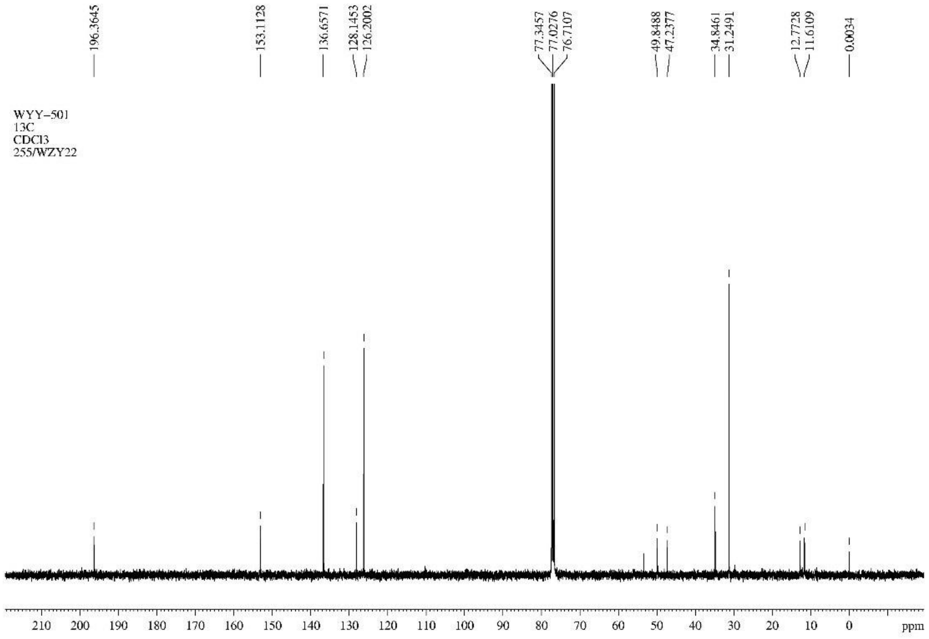

^{13}C NMR spectrum of compound **4d**

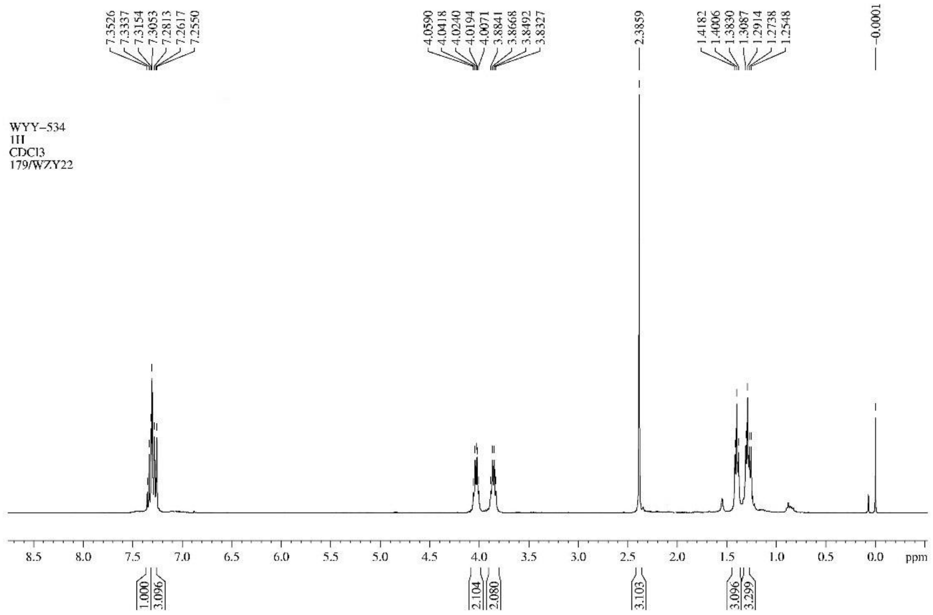

^{1}H NMR spectrum of compound **4e**

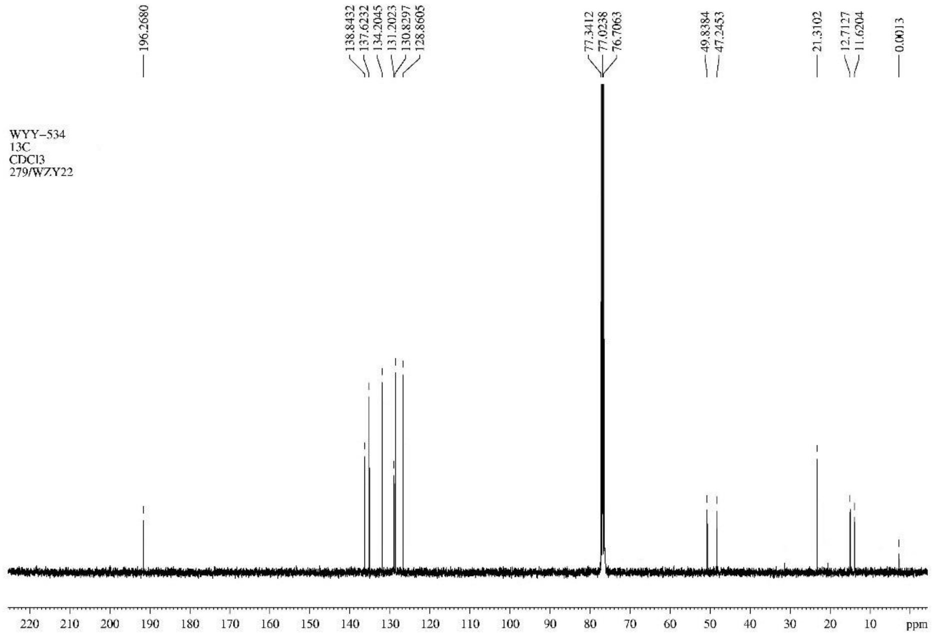

^{13}C NMR spectrum of compound **4e**

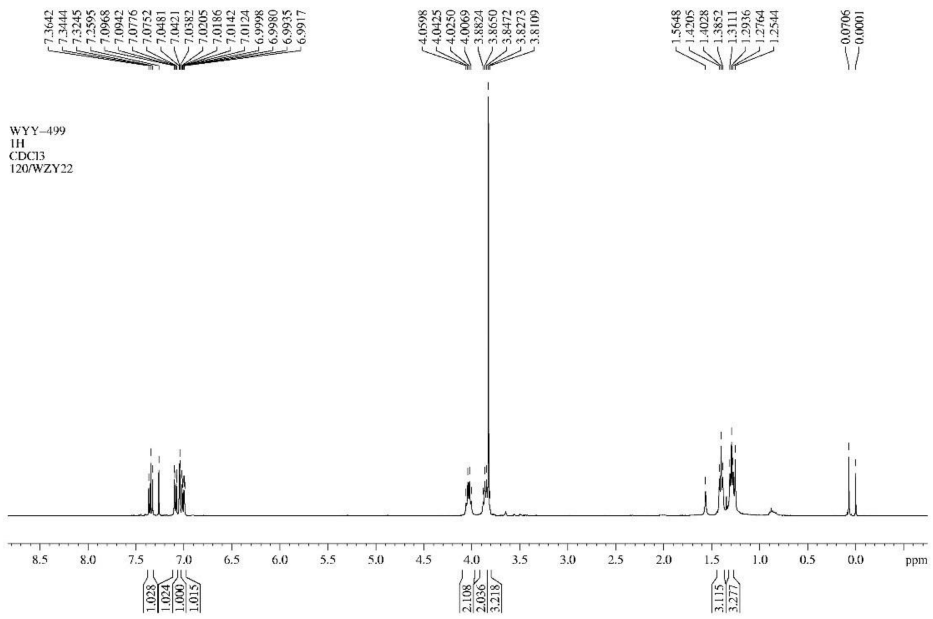

^{1}H NMR spectrum of compound **4f**

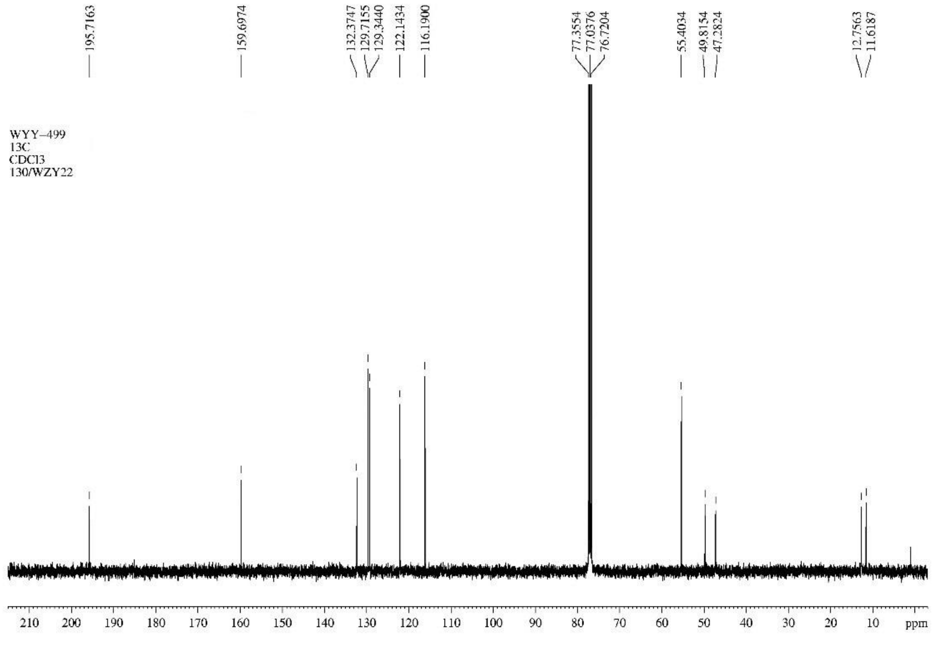

^{13}C NMR spectrum of compound **4f**

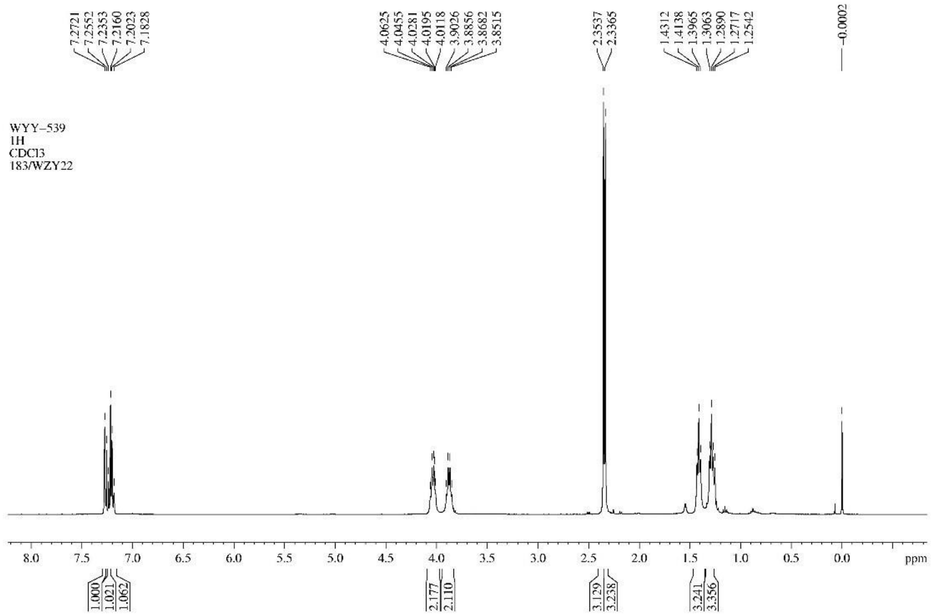

^{1}H NMR spectrum of compound **4g**

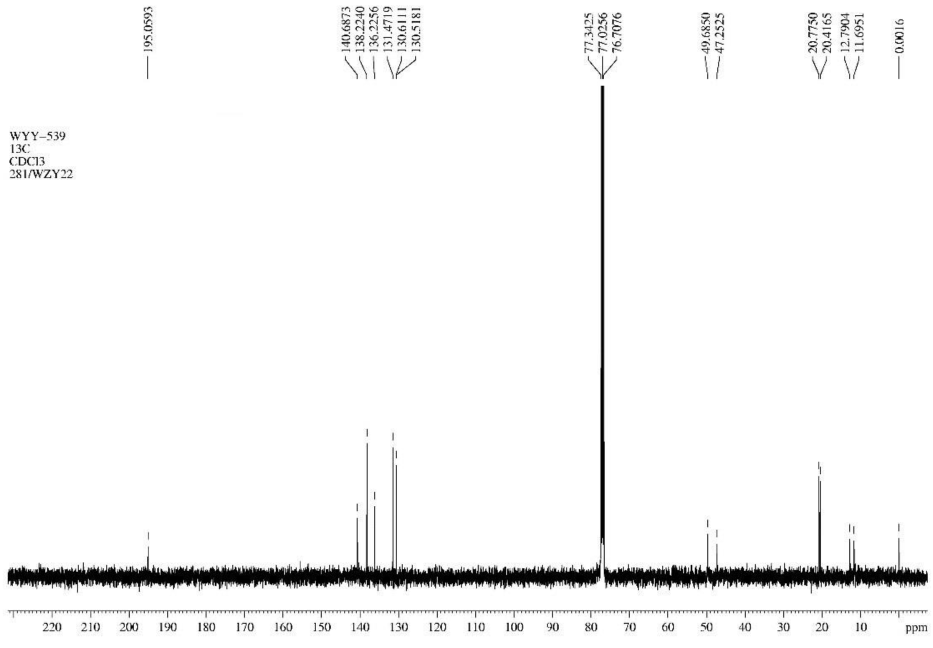

^{13}C NMR spectrum of compound **4g**

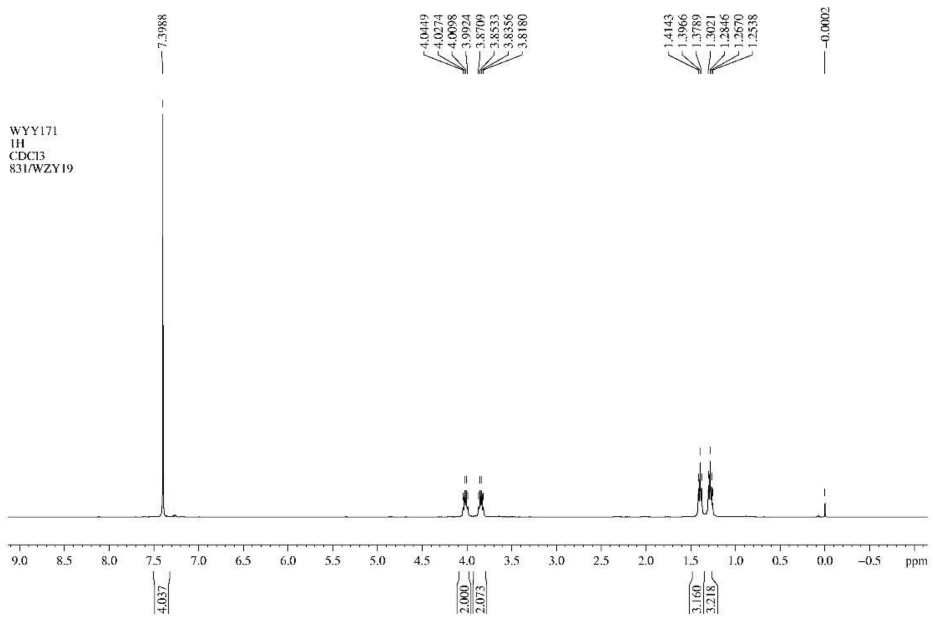

^{1}H NMR spectrum of compound **4h**

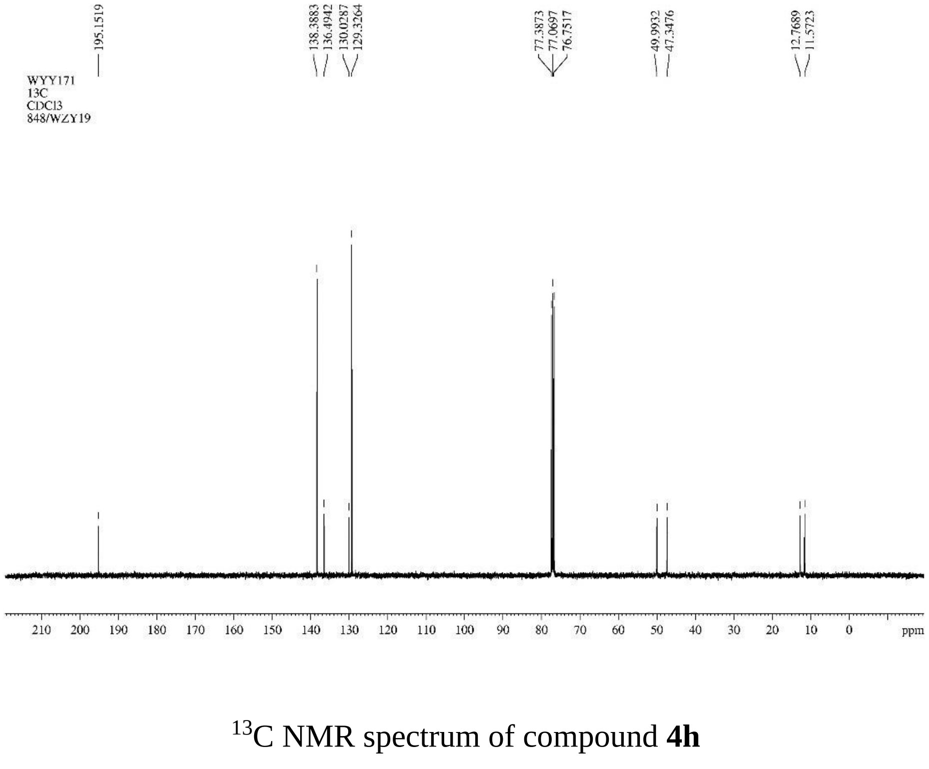

^{13}C NMR spectrum of compound **4h**

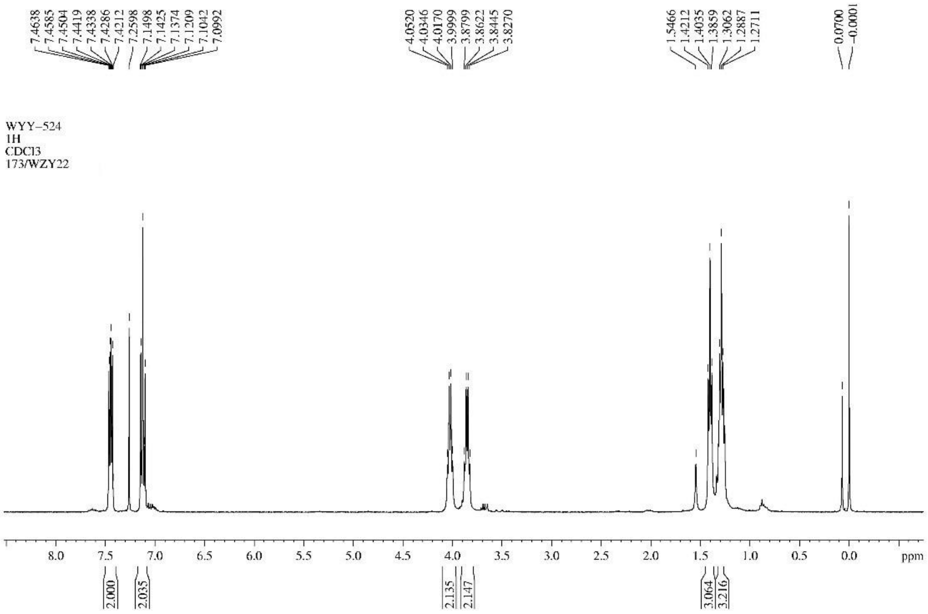

^{1}H NMR spectrum of compound **4i**

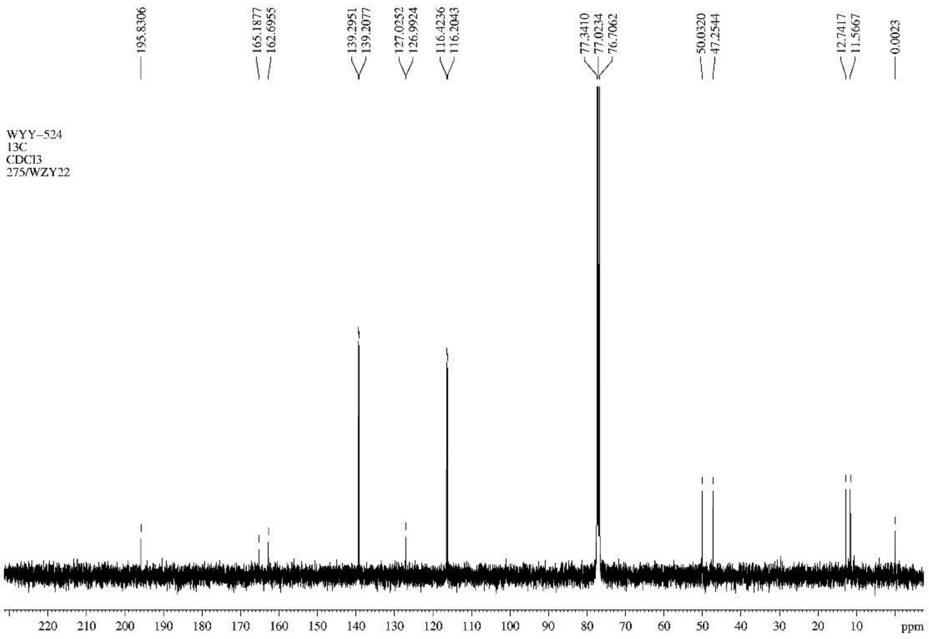

^{13}C NMR spectrum of compound **4i**

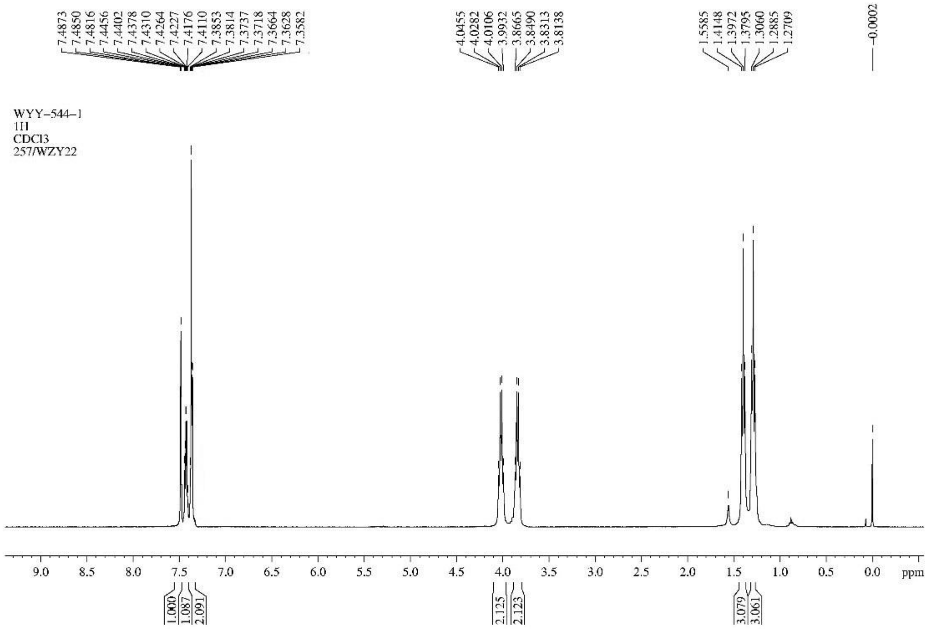

^{1}H NMR spectrum of compound **4j**

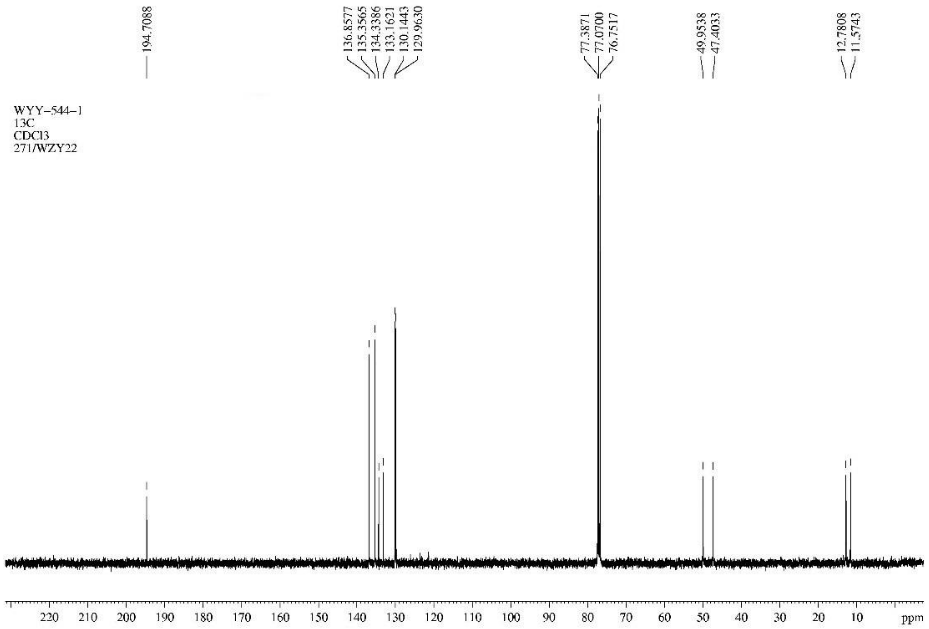

^{13}C NMR spectrum of compound **4j**

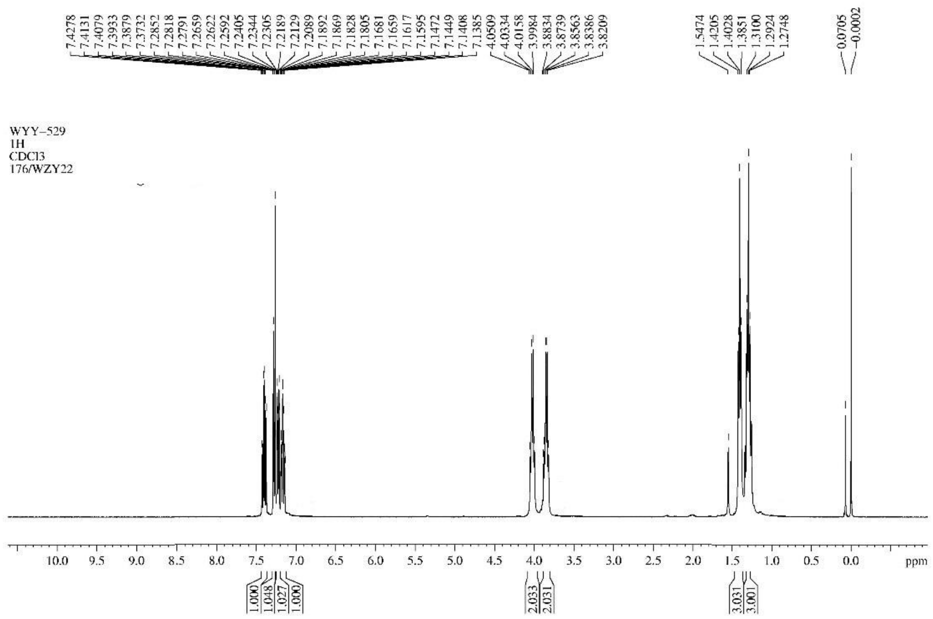

^{1}H NMR spectrum of compound **4k**

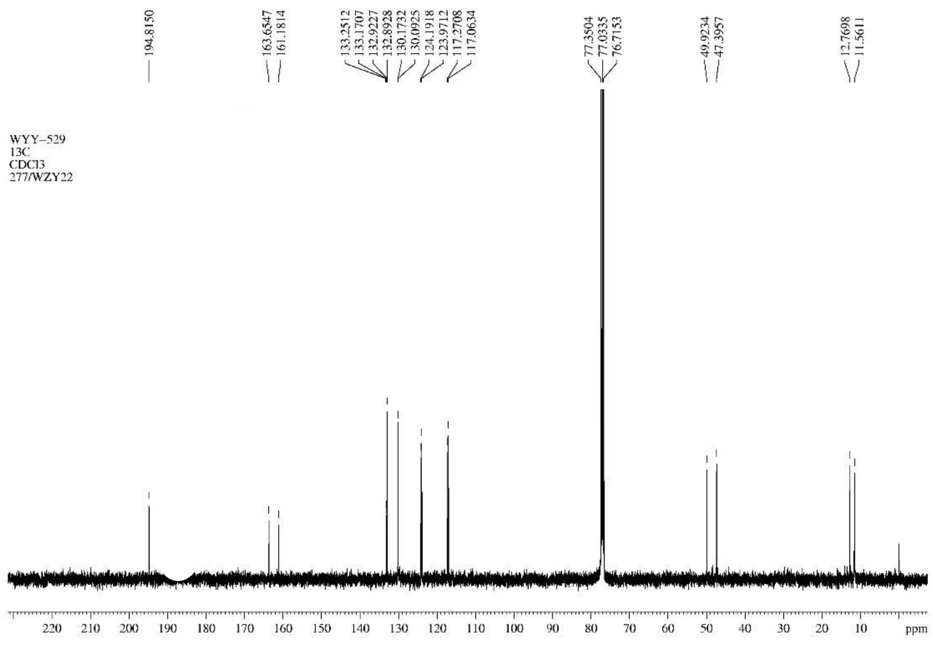

^{13}C NMR spectrum of compound **4k**

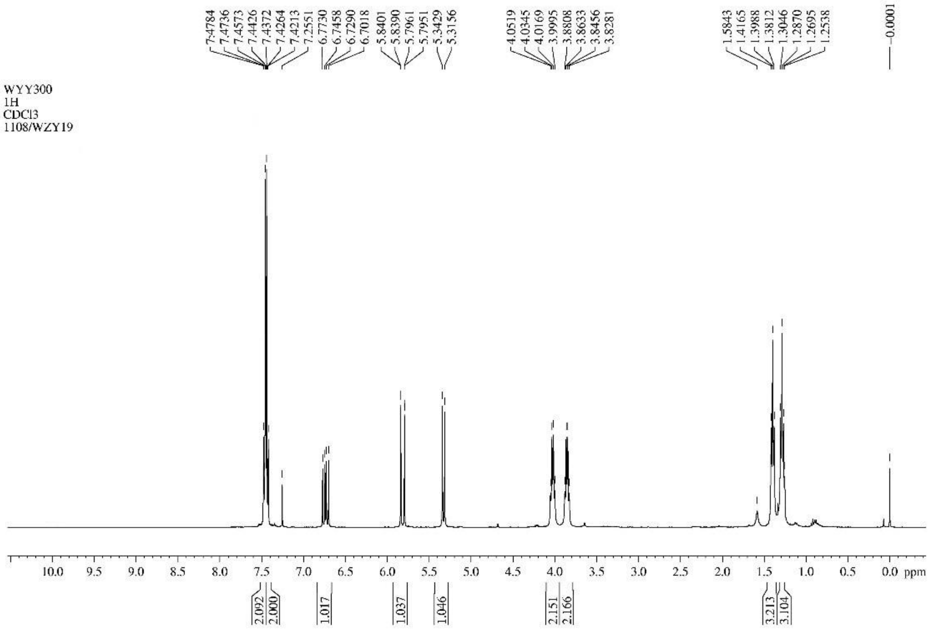

^{1}H NMR spectrum of compound **4l**

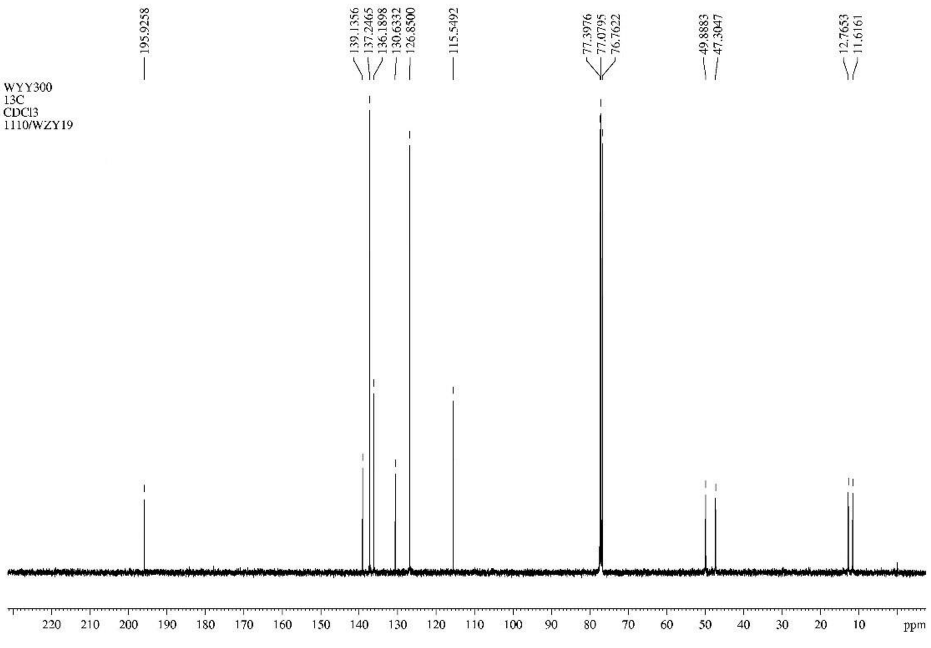

^{13}C NMR spectrum of compound **4l**

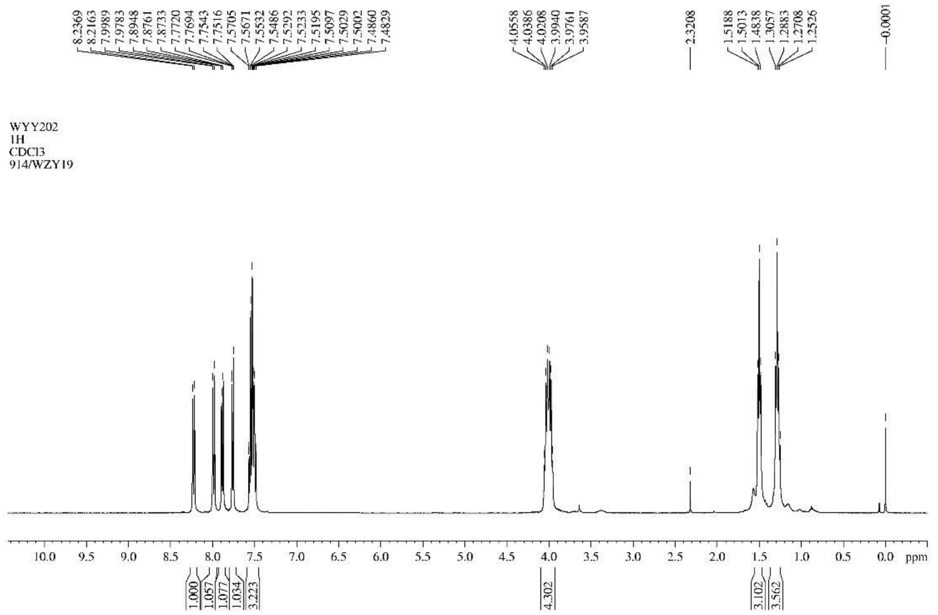

^{1}H NMR spectrum of compound **4m**

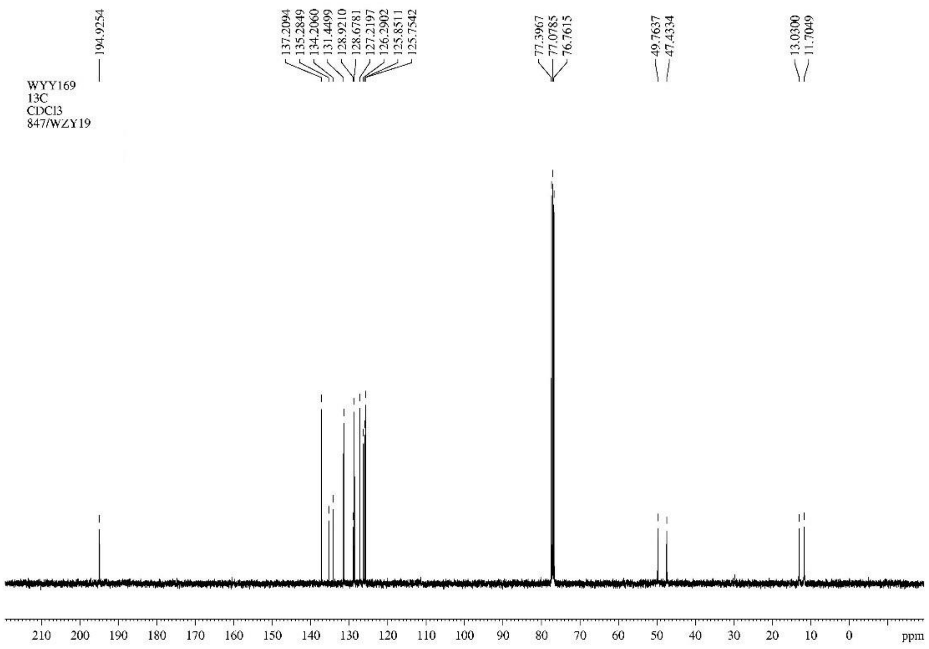

^{13}C NMR spectrum of compound **4m**

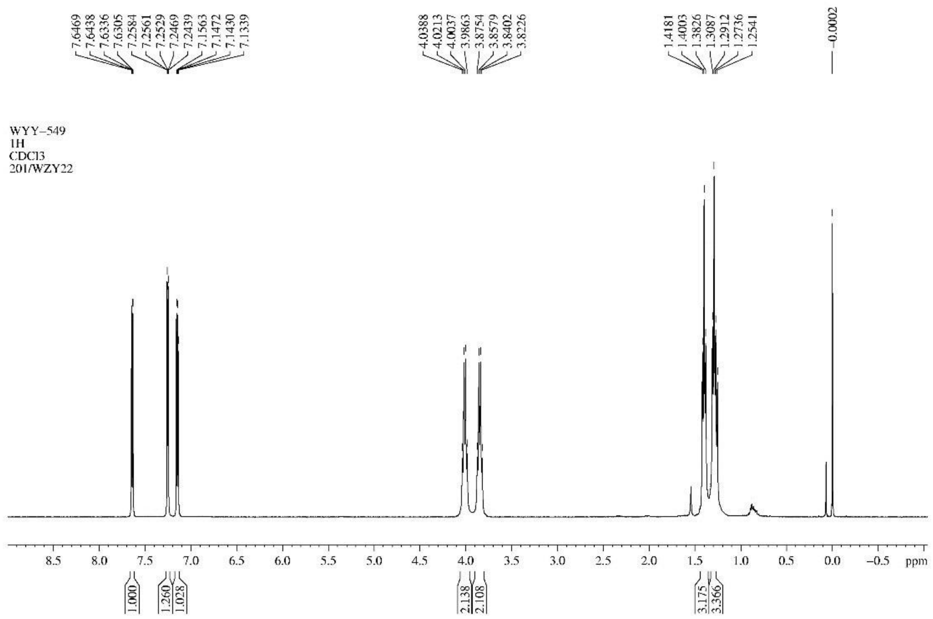

^{1}H NMR spectrum of compound **4n**

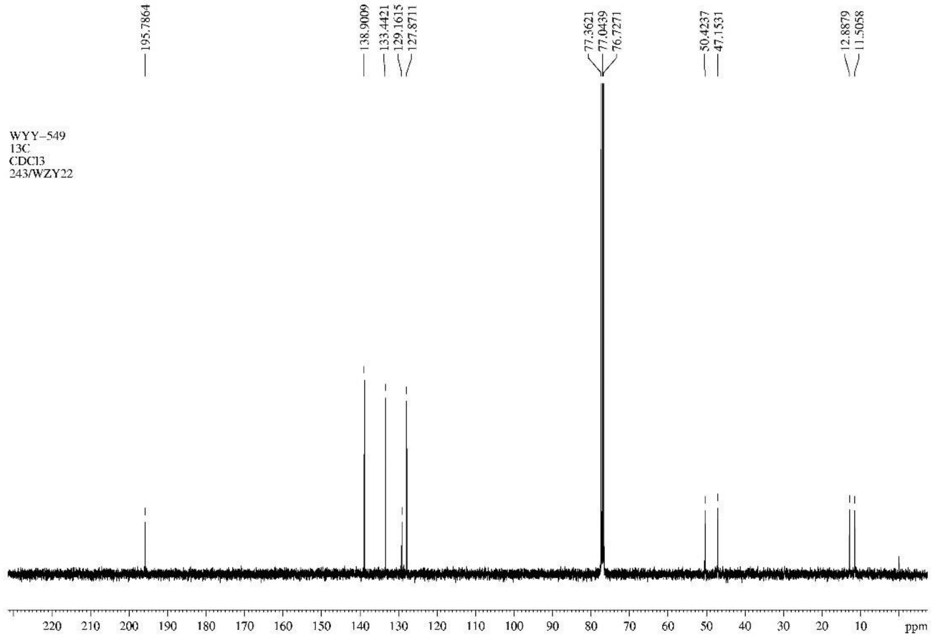

^{13}C NMR spectrum of compound **4n**

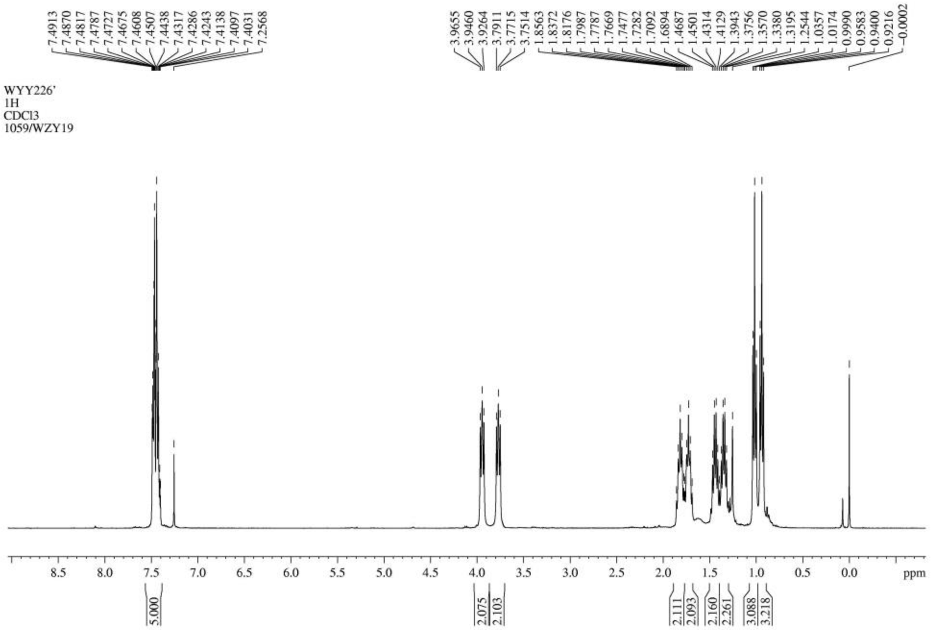

^{1}H NMR spectrum of compound **4o**

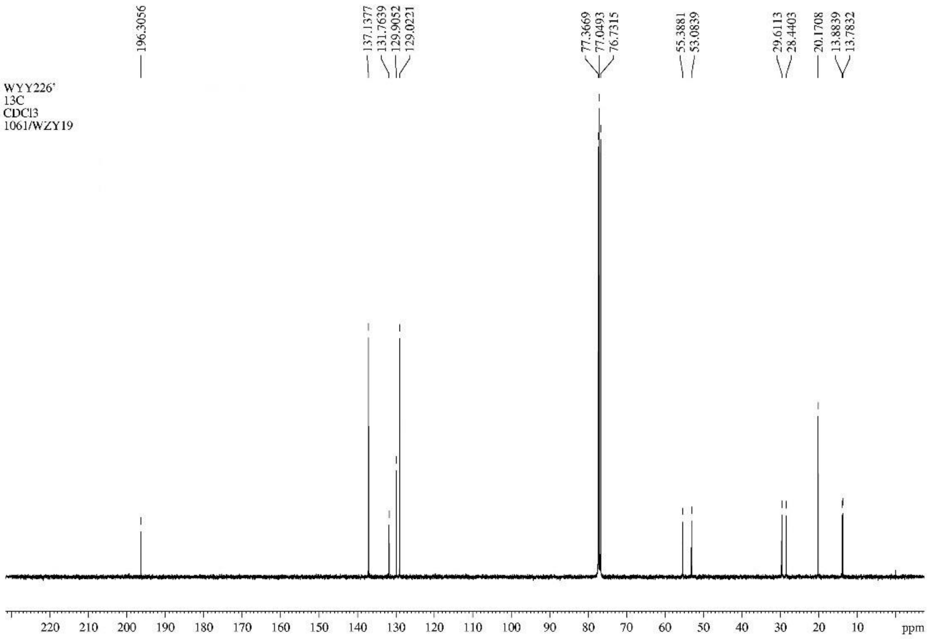

^{13}C NMR spectrum of compound **4o**

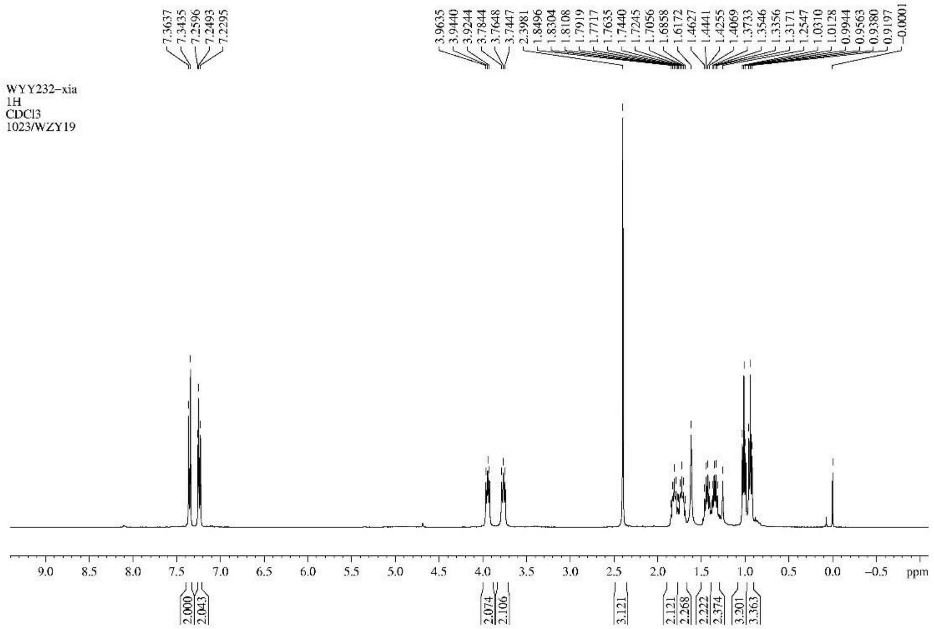

^{1}H NMR spectrum of compound **4p**

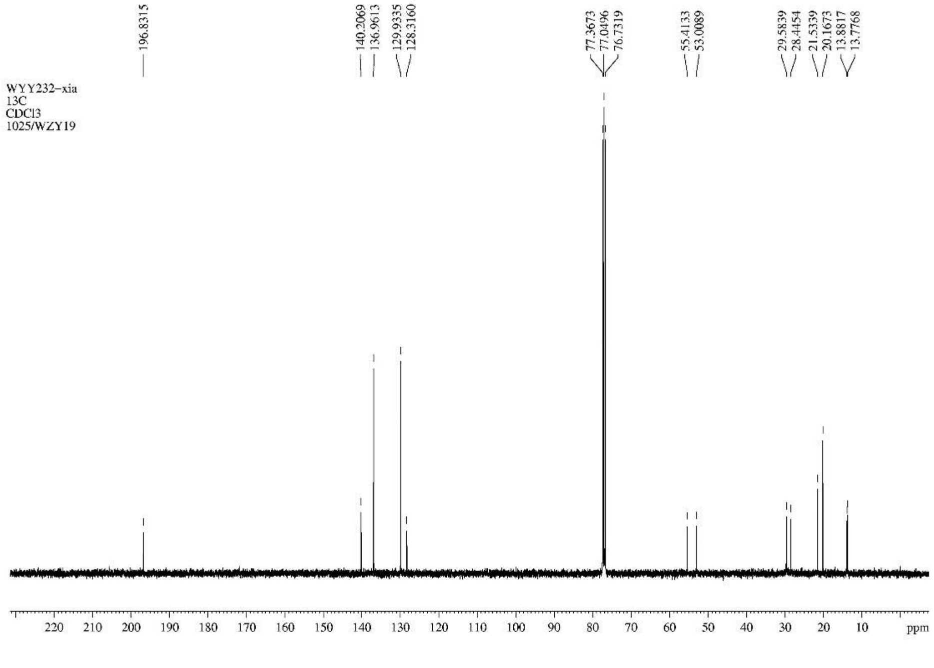

^{13}C NMR spectrum of compound **4p**

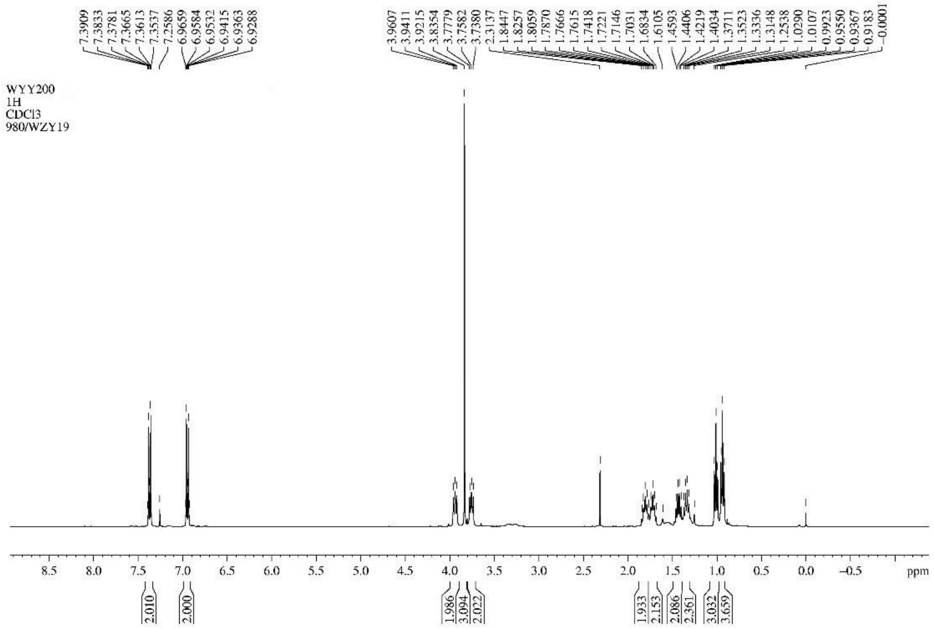

^{1}H NMR spectrum of compound **4q**

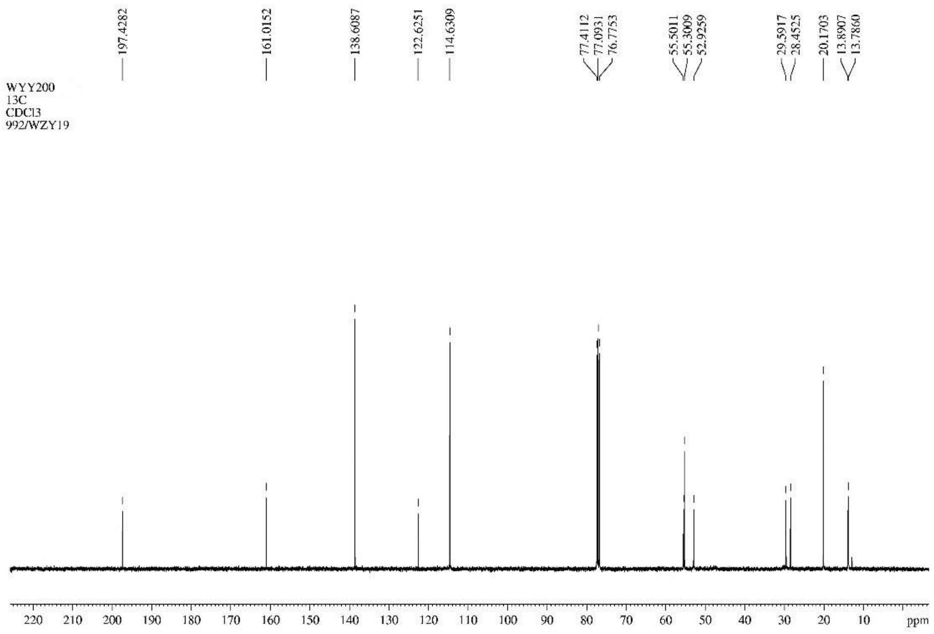

^{13}C NMR spectrum of compound **4q**

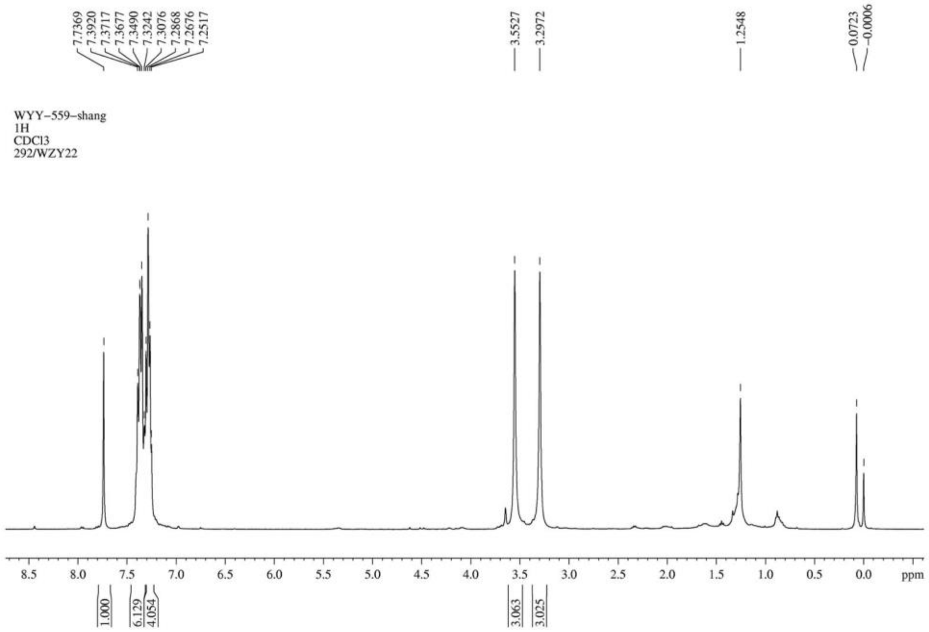

^{1}H NMR spectrum of compound **6**

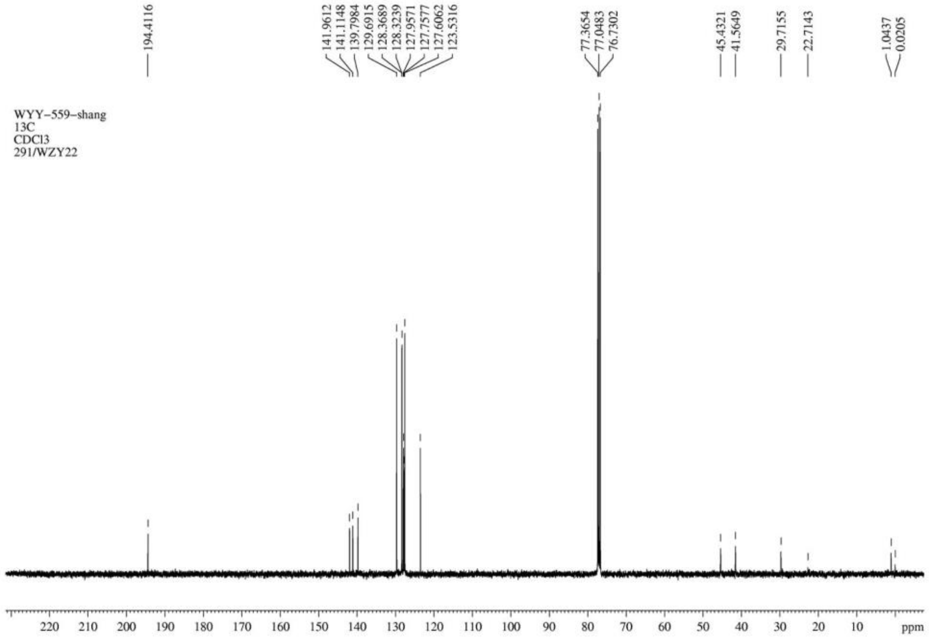

^{13}C NMR spectrum of compound **6**
